# Supplementary material for: Advanced monitoring and numerical modelling of the stability, safety and reliability indicators of the earthen dam of Songloulou (Cameroon)
Source: PLoS One. 2023 Oct 11;18(10):e0292804. doi: 10.1371/journal.pone.0292804 (PMC10566693; doi:10.1371/journal.pone.0292804)
Supplement: Attachment — Submitted filename: PONE-D-23-03305_reviewer.pdf [file pone.0292804.s001.pdf]

PLOS ONE

Advanced Monitoring and Numerical modeling of the Stability, Safety and Reliability Indicators of the Earthen Dam of Songloulou (Cameroon)

--Manuscript Draft--

Manuscript Number:	PONE-D-23-03305
Article Type:	Research Article
Full Title:	Advanced Monitoring and Numerical modeling of the<br>Stability, Safety and Reliability Indicators of the<br>Earthen Dam of Songloulou (Cameroon)
Short Title:	Advanced Monitoring and Numerical Modeling of the Stability, Safety and Reliability Indicators of the Dam
Corresponding Author:	ZOA AMBASSA<br>National Higher Polytechnic School of Douala<br>Douala, CAMEROON
Keywords:	Earth dam, artificial intelligence, piezometers, pendulums, ANFIS, HST, slope stability
Abstract:	For the determination of global stability after advanced long term monitoring, in this paper, we<br>used artificial intelligence for the analysis of water level and displacements data from the<br>Songloulou earth dam of Cameroon. Measurements of safety and reliability indicators follow<br>changes dictated by several reversible and irreversible phenomena like piezometric and<br>pendulums measurements. The results obtained over many years have confirmed the relevance<br>and robustness of models using artificial intelligence. We have simulated the behavior model<br>through piezometric and pendulum measurements of this dam more precisely the ANFIS<br>(Adaptive Neural Fuzzy Inference System) model, which combines the concept of artificial<br>neurons and that of fuzzy logic, has provided satisfactory results, given in the large amount of<br>data to be processed. The water level evolution is modeled using the ANFIS function integrated<br>in the MATLAB software and we compare it with the HST (Hydrostatic Season Time) method.<br>Afterwards, the stress state of the structure and shear slope stability are evaluated based on the<br>hydromechanical behavior using the GEOSTUDIO Finite Element calculation software. In this<br>case, the input parameters are: the hydraulic heads recorded in the piezometers and geotechnical<br>parameters of the dam. The modeling results in terms of displacement are perfectly consistent<br>with the displacement measurements. The horizontal displacement obtained in the<br>GEOSTUDIO model at the pendulums position is 80 mm and that of the pendulums is 70 mm<br>of average value. The safety factor for slope stability according water level of the 530 m is 1.7.
Order of Authors:	ZOA AMBASSA
	Jean Chills AMBA, Professor
	Merlin BODOL MOMHA

Powered by Editorial Manager® and ProduXion Manager® from Aries Systems Corporation

	Landry DJOPKOP KOUANANG
	Robert NZENGWA
	Pascal Adrien MBONGO
**Additional Information:**	
**Question**	**Response**
**Financial Disclosure**<br><br>Enter a financial disclosure statement that describes the sources of funding for the work included in this submission. Review the submission guidelines for detailed requirements. View published research articles from *PLOS ONE* for specific examples.<br><br>This statement is required for submission and **will appear in the published article** if the submission is accepted. Please make sure it is accurate.<br><br>**Unfunded studies**<br>Enter: *The author(s) received no specific funding for this work.*<br><br>**Funded studies**<br>Enter a statement with the following details:<br>• Initials of the authors who received each award<br>• Grant numbers awarded to each author<br>• The full name of each funder<br>• URL of each funder website<br>• Did the sponsors or funders play any role in the study design, data collection and analysis, decision to publish, or preparation of the manuscript?<br>• **NO** - Include this sentence at the end of your statement: *The funders had no role in study design, data collection and analysis, decision to publish, or preparation of the manuscript.*<br>• **YES** - Specify the role(s) played.<br><br>* typeset	No
**Competing Interests**<br><br>Use the instructions below to enter a competing interest statement for this submission. On behalf of all authors, disclose any competing interests that could be perceived to bias this	No

work—acknowledging all financial support and any other relevant financial or non-financial competing interests.<br><br>This statement is required for submission and **will appear in the published article** if the submission is accepted. Please make sure it is accurate and that any funding sources listed in your Funding Information later in the submission form are also declared in your Financial Disclosure statement.<br><br>View published research articles from *PLOS ONE* for specific examples.<br><br>**NO authors have competing interests**<br><br>Enter: *The authors have declared that no competing interests exist.*<br><br>**Authors with competing interests**<br><br>Enter competing interest details beginning with this statement:<br><br>*I have read the journal's policy and the authors of this manuscript have the following competing interests: [insert competing interests here]*<br><br>* typeset	
**Ethics Statement**<br><br>Enter an ethics statement for this submission. This statement is required if the study involved:<br><br>• Human participants<br>• Human specimens or tissue<br>• Vertebrate animals or cephalopods<br>• Vertebrate embryos or tissues<br>• Field research<br><br>Write "N/A" if the submission does not require an ethics statement.	Human participants

General guidance is provided below. Consult the submission guidelines for detailed instructions. **Make sure that all information entered here is included in the Methods section of the manuscript.**<br><br>**Format for specific study types**<br><br>**Human Subject Research (involving human participants and/or tissue)**<br>• Give the name of the institutional review board or ethics committee that approved the study<br>• Include the approval number and/or a statement indicating approval of this research<br>• Indicate the form of consent obtained (written/oral) or the reason that consent was not obtained (e.g. the data were analyzed anonymously)<br><br>**Animal Research (involving vertebrate animals, embryos or tissues)**<br>• Provide the name of the Institutional Animal Care and Use Committee (IACUC) or other relevant ethics board that reviewed the study protocol, and indicate whether they approved this research or granted a formal waiver of ethical approval<br>• Include an approval number if one was obtained<br>• If the study involved *non-human primates*, add *additional details* about animal welfare and steps taken to ameliorate suffering<br>• If anesthesia, euthanasia, or any kind of animal sacrifice is part of the study, include briefly which substances and/or methods were applied<br><br>**Field Research**<br><br>Include the following details if this study involves the collection of plant, animal, or other materials from a natural setting:<br>• Field permit number<br>• Name of the institution or relevant body that granted permission	
**Data Availability**<br><br>Authors are required to make all data underlying the findings described fully	No - some restrictions will apply

Powered by Editorial Manager® and ProduXion Manager® from Aries Systems Corporation

available, without restriction, and from the time of publication. PLOS allows rare exceptions to address legal and ethical concerns. See the PLOS Data Policy and FAQ for detailed information.<br><br>A Data Availability Statement describing where the data can be found is required at submission. Your answers to this question constitute the Data Availability Statement and **will be published in the article**, if accepted.<br><br>**Important:** Stating 'data available on request from the author' is not sufficient. If your data are only available upon request, select 'No' for the first question and explain your exceptional situation in the text box.<br><br>Do the authors confirm that all data underlying the findings described in their manuscript are fully available without restriction?	
**Describe where the data may be found in full sentences. If you are copying our sample text, replace any instances of XXX with the appropriate details.**<br><br>• If the data are **held or will be held in a public repository**, include URLs, accession numbers or DOIs. If this information will only be available after acceptance, indicate this by ticking the box below. For example: *All XXX files are available from the XXX database (accession number(s) XXX, XXX.*).<br>• If the data are all contained **within the manuscript and/or Supporting Information files**, enter the following: *All relevant data are within the manuscript and its Supporting Information files.*<br>• If neither of these applies but you are able to provide **details of access elsewhere**, with or without limitations, please do so. For example:<br><br>*Data cannot be shared publicly because of [XXX]. Data are available from the XXX Institutional Data Access / Ethics*	This test is appropriate if the data are owned by à third party and authors do not have permission to share the data

Committee (contact via XXX) for researchers who meet the criteria for access to confidential data.<br><br>*The data underlying the results presented in the study are available from (include the name of the third party and contact information or URL).*<br>• This text is appropriate if the data are owned by a third party and authors do not have permission to share the data.<br><br>* typeset	
Additional data availability information:	Tick here if the URLs/accession numbers/DOIs will be available only after acceptance of the manuscript for publication so that we can ensure their inclusion before publication.; Tick here if your circumstances are not covered by the questions above and you need the journal's help to make your data available.

Manuscript
Click here to access/download;Manuscript;Advanced Monitoring dam Plos one.doc

Advanced Monitoring and Numerical modeling of the Stability, Safety and Reliability Indicators of the Earthen Dam of Songloulou (Cameroon)

Zoa Ambassa[1,2] *, Jean Chills Amba [1,2], Merlin Bodol Momha[1], Landry Djopkop Kouanang[1,2], Robert Nzengwa[1,2], Pascal Adrien Mbongo[3]

1 Laboratory of Energy Modeling Materials and Methods (E3M), National Higher Polytechnic School of Douala, University of Douala, P.O. Box: 2701 Douala, Cameroon, **2** Department of Civil Engineering, National Higher Polytechnic School of Douala, University of Douala, Cameroon, **3** The Energy of Cameroon (ENEO), Douala, Cameroon

*daniel.zoa77@yahoo.fr

Abstract

For the determination of global stability after advanced long term monitoring, in this paper, we used artificial intelligence for the analysis of water level and displacements data from the Songloulou earth dam of Cameroon. Measurements of safety and reliability indicators follow changes dictated by several reversible and irreversible phenomena like piezometric and pendulums measurements. The results obtained over many years have confirmed the relevance and robustness of models using artificial intelligence. We have simulated the behavior model through piezometric and pendulum measurements of this dam more precisely the ANFIS (Adaptive Neural Fuzzy Inference System) model, which combines the concept of artificial neurons and that of fuzzy logic, has provided satisfactory results, given in the large amount of data to be processed. The water level evolution is modeled using the ANFIS function integrated in the MATLAB software and we compare it with the HST (Hydrostatic Season Time) method. Afterwards, the stress state of the structure and shear slope stability are evaluated based on the hydromechanical behavior using the GEOSTUDIO Finite Element calculation software. In this case, the input parameters are: the hydraulic heads recorded in the piezometers and geotechnical parameters of the dam. The modeling results in terms of displacement are perfectly consistent with the displacement measurements. The horizontal displacement obtained in the GEOSTUDIO model at the pendulums position is 80 mm and that of the pendulums is 70 mm of average value. The safety factor for slope stability according water level of the 530 m is 1.7.

Keywords: Earth dam, artificial intelligence, piezometers, pendulums, ANFIS, HST, Slope stability.

1. Introduction

The evaluation and analysis of the monitoring data of a dam during its lifetime make it possible to process the influence of external parameters on these measurements [1-4]. Indeed, the variations in measurements recorded on the dam's result from a combination of immediately inseparable factors, whatever the nature and the sophistication of the monitoring instruments used [5-9]. Three factors are preponderant; two of them are reversible if the stability or resistance limits are not reached: the hydrostatic conditions, i.e. the water level in the reservoir and the climatic conditions (temperature, rain, water content) [5-9]. The third irreversible is related to the age of the dam [5-9]. A real analysis of the structure assumes that we can follow the evolution of its behavior over time, after deducting any other variation [1-9]. The first step consists in displaying the measurements of each instrument on a graph adapted to the phenomenon to be analyzed: in general, several years for the mechanical quantities, and according to the retention level for the hydraulic quantities, by also showing the threshold to check that there is no read error [5-14]. This first level of analysis most often makes it possible to detect, at least qualitatively, sudden and even slow irreversible variations, in particular when there are few explanatory factors (restraint at an almost constant level, measurements not subject to seasonal influences). However, this level of analysis turns out to be insufficient, in particular for the largest dams, as soon as one wishes to access numerical values within reasonable time or when several explanatory factors coexist; more sophisticated statistical models are used. Moreover, this level of analysis may prove to be insufficient when one wishes to access numerical values. For this category of structures, the importance of rapid processing of auscultation data considered essential for the analysis of overall or partial stability of the dam is obvious [15-17]. It is advisable to use deterministic models or even statistical models to improve the reliability of the measurements.

Regarding deterministic models, finite element calculation models are commonly used at the dam project stage [15]. Indeed, subject to a good knowledge of the parameters of material behavior laws, they make it possible to reliably represent the deformations and stresses in the embankment in different project situations. They also make it possible to assess the state of stress during the life of the structure, especially in the context of safety reassessment studies [18-25]. The limits of these approach lies in the fact that the calculations are quite laborious

when one wishes to model the regular monitoring of the structure and, above all, they do not easily allow the consideration of the complexity of the factors which influence the nonlinear behavior and the heterogeneities of soil, rock and concrete materials. However, the statistical analysis methods for auscultation measurements seem more advantageous because they make it possible to separate the respective influences of several explanatory factors introduced into the model. Among them, we can cite the ‘HST’ method which was originally developed for the pendulums of arch dams (Carrere). The main disadvantage related to the HST method, which is based on multiple linear regression, is the interdependence between the variables H and S and the explained value is approximated to a maximum of 85%. In addition, given the aging of the structure, the coefficients of the explanatory variables come to change, which requires recalibrating the model after a certain time. The displacements measured on either side of the dam and the uplifts measured in the dams at the level of the concrete-rock contact and upstream are generally strongly influenced by the upstream coast. The data shows that this hydrostatic effect is not the same in the dry season and in the wet season. The zone in which the uplifts are measured is a "fractured zone" and its state of fracturing depends on the position of the dam: in the dry season, with the expansion of the concrete, the dam assumes an upstream position and the cracking at the substructure tends to close. Conversely, in the rainy season, with the contraction of the concrete, the dam is positioned further downstream and cracking at the upstream toe is greater. This effect is not included in the HST model which considers a hydrostatic effect averaged over all the seasons. To overcome this drawback, we use in this paper neural networks which have the ability to create differentiated hydrostatic effects according to the seasons.

On the other hand, for the particular case of hydraulic measurements (piezometry and flow rates), a hysteresis effect is commonly observed when representing the value of the pore pressure in the core or an earth dam foundation, as a function of the coast of restraint. Indeed, the path described when going up the body of water is not the same as the way down. Bonelli and collective [1, 6-8] explain this phenomenon by the fact that the soil capacity is never null, because the presence of dissolved or occluded air, even for compacted soils close to saturation. The model uses two parameters to characterize the maximum amplitude that the hydraulic head can reach in the piezometer under the effect of the stress and the delay effect. The proposed relationship is consistent with the modeling of the delay from a Kelvin-Voigt model on non-ageing material. With regard to the explanatory variables used, the Exponential_IRF model can only serve as a complement to a more global one. Thus,

compared to the models described above, taking into account the delayed response seems more suitable for analyzing data when there is a difference between the solicitation and the response measured by the monitoring instrument. Despite this consideration of the delayed response, it can be seen that the modeling of the evolution of the measured parameters does not agree perfectly with the real data. This delayed effect is more suitable for embankment dams but for concrete dams the modeling seems more delicate given the number of parameters to be considered [6-7].

In this paper, an analysis of auscultation devices based on artificial neural networks is implemented. The method adapts well to variations in the parameters influencing the measurement tools and does not require ongoing recalibration during the lifetime of the structure. Unlike the HST method based on data recorded during a period (analysis period), neural networks use the entire data recorded during operation and improve their efficiency over time. Indeed, the neural network method makes it possible to model nonlinearities based on the use of the properties of time series [26]. These non-linearities are closely linked to the evolution of geotechnical parameters, environmental parameters, as well as structural parameters of the dam.

2. Presentation of structures and context of Research

2.1 Presentation of the Songloulou hydroelectric earthen dam

The Songloulou large earthen dam (Fig 1) is located in the Littoral region of Cameroon, towards Babimbi, near Massok. It was implanted on the Sanaga river. With its vast watershed representing more than 25% of the total area of the country and its many rapids. The Sanaga is largest river in Cameroon and constitutes a reservoir of hydroelectric energy of the first order. The main dam, leveled at height 530 m and has a central sealing core in compacted lateritic clay, separated from the upstream and downstream rock fill by sand and gravel filters. A riprap zone with large rock fill provides anti-swing protection on the upstream face and a 9 m wide crest. The spillway is calculated to evacuate an exceptional flood of 10,000 m^3/s below elevation 528.50 m with a blocked valve. It has seven passes closed by segment gates 14 m wide and 17 m high, controlled by winches and chains. The passes, whose sill is at elevation 511 m, are separated by pillars 4.5 m thick, which of 35 m, is made up of eight sluices13.5 m wide. The intake dam, with a maximum height of 35 m, is made up of eight sluices13.5 m wide separated by buttresses, a connecting gravity wall to the left bank and connecting buttress structure at the spillway. The openings of the four intakes on the left bank

are equipped with large screens, cleaned by a bar screen rolling on the crown of the structure at elevation 530.

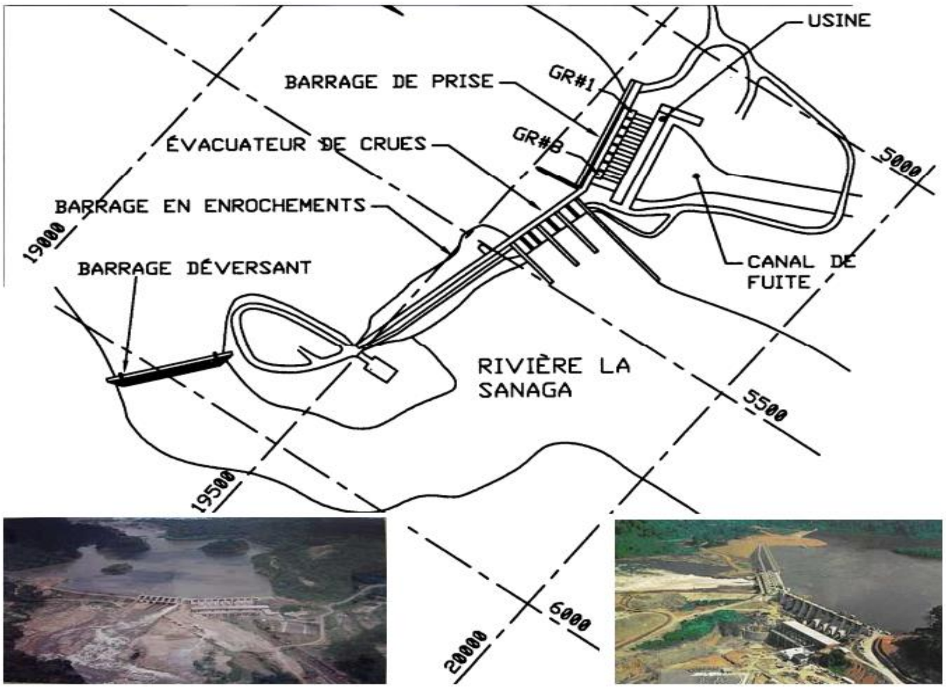

Fig 1. General view of the Songloulou hydroelectric development

2.2 Context and issue

Concrete swelling phenomena were identified six years after the commissioning of the first units (1981) by reducing the clearances between the fixed and moving parts of the turbines and alternators. These swellings had various consequences, making the operation and safety of the structures difficult. Studies have been undertaken since 1991, aimed at evaluating and improving the safety of structures, as well as a series of interventions to counter the effects of swelling on the equipment with increased auscultation for the monitoring of the structures [27]. On the Songloulou development, monitoring data is regularly collected but the site has no means of analyzing this data automatically. Analyzing these auscultation data raises questions about the effective technique to use given the advantages and limitations of monitoring techniques. The objective of this paper is to analyze the auscultation data of the Songloulou hydroelectric dam using artificial intelligence and to propose a method of prediction and assistance in the global analysis of said data for the operation of the structure safe.

3. Materials and method of monitoring data analysis

The Songloulou hydroelectric dam has within it 12 pendulums to measure displacements, 40 piezometers for pressures and more than 200 other monitoring devices. In this paper we are interested in the analysis of sub-pressures and horizontal displacements on concrete and earth structures, that is to say the intake dam and the spillway dam. The layout of the piezometric devices in these works is illustrated in Fig 2 to 5.

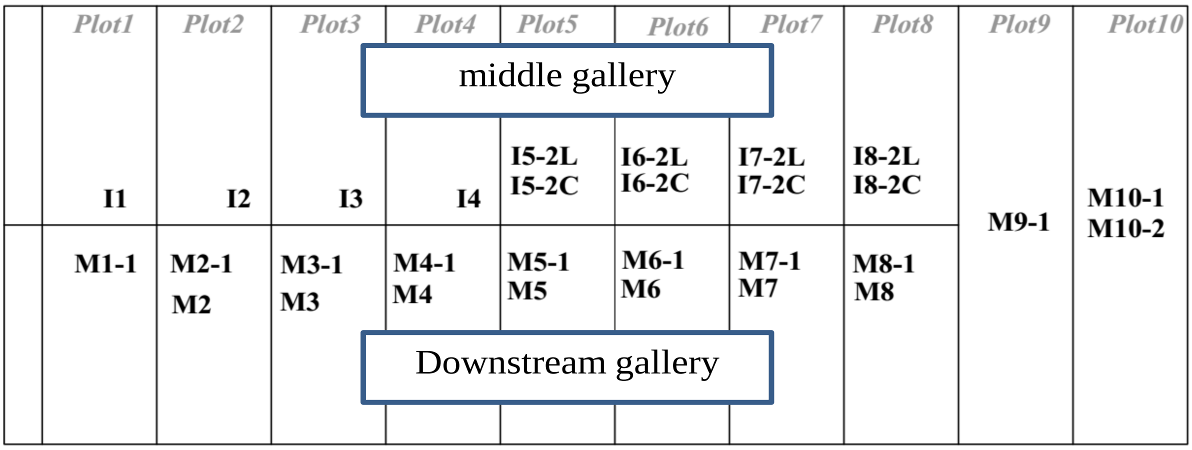

Fig 2. Position of the piezometers at the Songloulou water intake dam

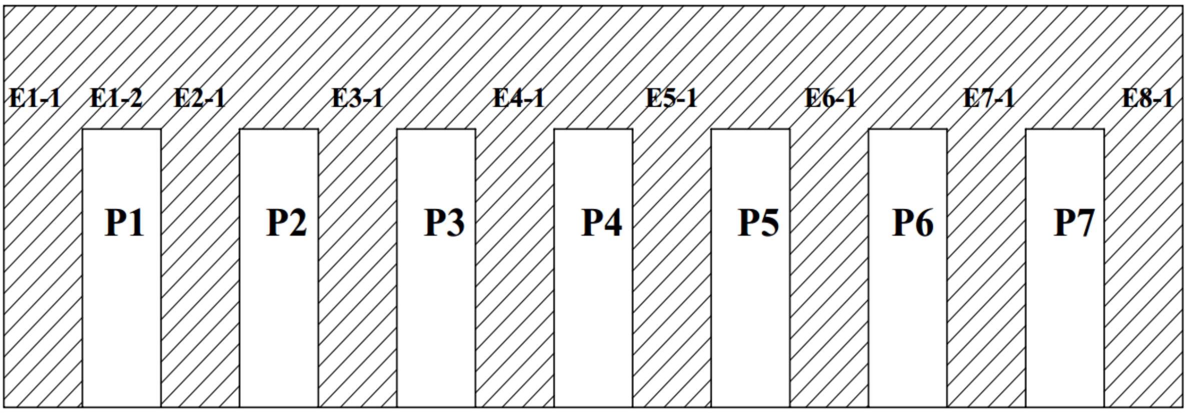

Fig 3. Position of the piezometers at the Songloulou evacuator dam

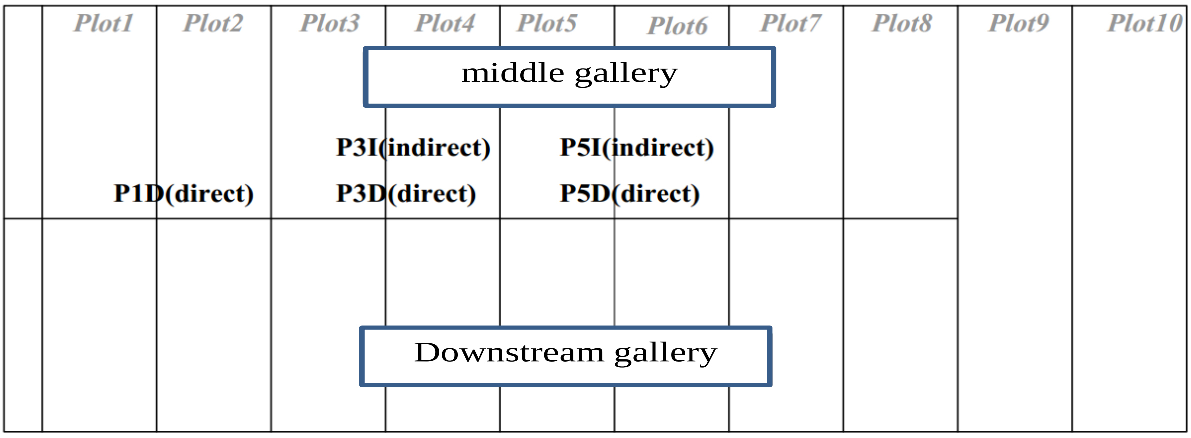

Fig 4. Position of the pendulums at the intake dam of the Songloulou hydroelectric development

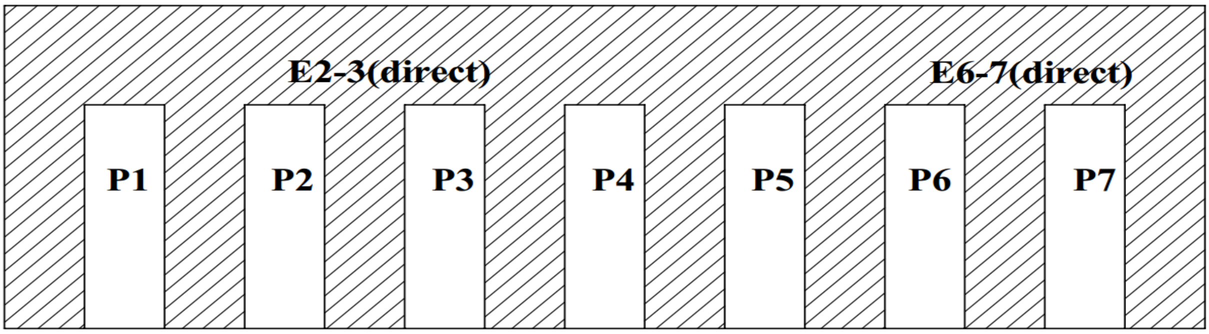

Fig 5. Position of the pendulums at the spillway dam of the Songloulou hydroelectric facility

The methodology consists of analyzing this quantity of data collected on the site at regular intervals for more than a decade using artificial intelligence models. Several research works have been carried out around the world using these techniques to guarantee the reliability and safety of dams [26-34]. On the intake dam, 30 piezometers are usable out of the 31 installed. On the gallery spillway dam, 09 piezometers can be used. These structures are subject to regular monitoring. We are interested in the behavior of this dam during the period from 1997 to 2019 for the pendulum data and from 2008 to 2019 for the piezometric data.

3.1. Calibration and operation of the fuzzy neuron tool

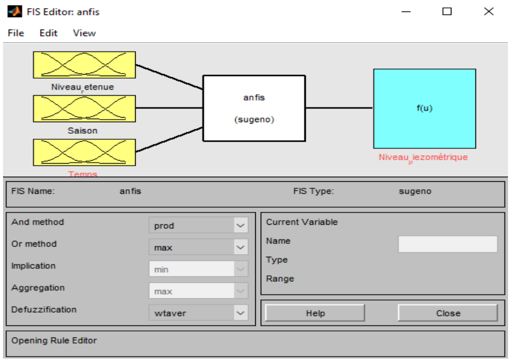

Fig 6 -a. Determination of inputs and outputs

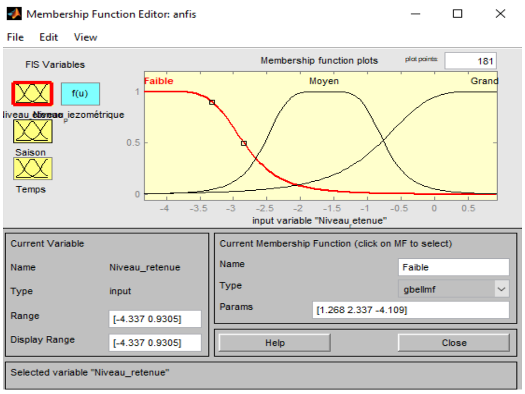

Fig 6 -b. Variable membership functions

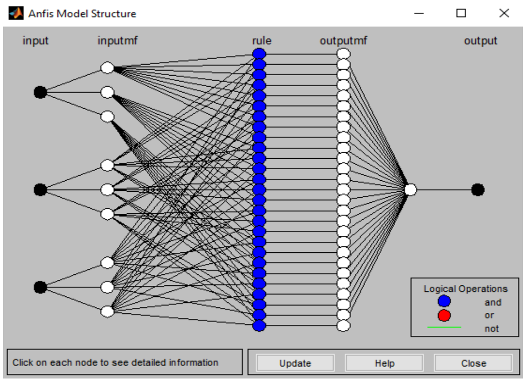

Fig 6 -c. Architecture of the ANFIS network

For the realization of this work, we use Matlab software [35], in the applications (APPS) that this software offers, in the "CONTROL SYSTEM DESIGN AND ANALYSIS" part, the "Neuro-fuzzy Designer" application, the main tool of our programming. For our model (Fig 6) we will use three explanatory variables as input : the level of the reservoir (h), the season (s) and the time (t). The output will represent the estimated behavior o the piezometer.

3.2. Network of the parameters of the fuzzy neuron method

All the details used in this work are developed in the scientific and technical documents published by the referenced authors [9, 26, 28-34]. It should be recalled here that, the fuzzy neuron method is commonly used for the analysis of the behavior of civil engineering and hydraulic structures. We refer to some recent works that have used this technique [36-42] with satisfactory results.

Table 1. Presentation of the piezometer network

**Number of entry**	**3**
Membership functions	Bellshape 3 for entrance
T-norm	Product
Type of inference	Sugeno
Number of neuron	67
Number of premise parameters	27= 3*3
Number of consequent parameters	108=27*4
Total number of parameters	135
Total number of training data	132
Learning algorithm	Gradient back propagation and least squares method
Number of fuzzy rulers	27

Table 1 describes the neural network developed, it can be interpreted as follows: the number of entries represents the number of explanatory variables which are here the level of the reservoir, the season and the time; the membership fuction is chosen to describe the phenomenon, given that the curves are not affine and go jagged, we have opted for the Bellshape fuction. The type of inference chosen in this study is Sugeno because the membership fuctions are precise values.

4. Analysis of the HTS model and that of the fyzzy neural network

4.1 Intake dam : exploitation of piezometric data

4.1.1 Use of piezometer I1

Table 2 presents a summary of the new values of the membership fuction parameters of the piezometer I1 using Bellshape type membership fuctions. From the ANFIS model (Adaptive Neuro Fuzzy Inference System) the results of the piezometer I1 are given.

Table 2. Final parameters of the membership functions of the piezometer I1

final function	weak			average			hight		
	**ai**	**bi**	**Ci**	**ai**	**bi**	**ci**	**ai**	**bi**	**ci**
level	1.0947	2.3585	-4.2974	0.4738	2.5804	-1.3234	1.8114	1.6035	0.9052
season	1.5757	1.182	-0.2852	1.1378	3.3772	2.2213	1.6374	3.3772	2.2213
time	2.7784	2.3521	0.2099	1.5173	3.8164	5.2822	3.8372	3.3488	8.9724

Table 3. Coefficient of final parameters

function	Parameters				
	**Coefficient of the variable level retained: n**	**of the variable Season: s**	**of the variable Time: t**	**b**	w_i
$out1\_f_{1}$	-66.86201	14.66457	103.99512	28.52094	0.000718018
$out1\_f_{2}$	-13.67075	80.16198	118.95478	32.38700	0.002916573
$out1\_f_{3}$	26.12609	52.01292	37.84743	10.14450	0.003682125
$out1\_f_{4}$	-60.08609	34.65492	62.39650	16.05303	0.000398601
$out1\_f_{5}$	-21.82759	32.43701	58.24856	16.96844	0.001619106
$out1\_f_{6}$	12.12196	40.67423	55.84260	10.86692	0.002044095
$out1\_f_{7}$	-1.22492	-20.75657	-8.41647	-0.06685	0.000799131
$out1\_f_{8}$	-8.96193	4.18536	2.33630	9.01214	0.003246051
$out1\_f_{9}$	-12.70787	-0.77933	43.79042	7.22182	0.004098085
$out1\_f_{10}$	-78.95026	181.33469	71.64195	215.4254	0.000748617
$out1\_f_{11}$	3.25957	-29.03033	125.71012	15.11556	0.003040863
$out1\_f_{12}$	13.82312	55.21798	37.42386	-19.59825	0.00383904
$out1\_f_{13}$	2.05086	-124.93501	191.85175	99.11219	0.000415587
$out1\_f_{14}$	34.83082	72.43588	89.51021	88.88520	0.001688105
$out1\_f_{15}$	-77.30770	-142.24788	55.84266	16.87389	0.002131204
$out1\_f_{16}$	-12.24695	50.66410	115.60437	20.02558	0.000833186
$out1\_f_{17}$	-8.64564	-18.49168	109.95610	72.26042	0.003384382
$out1\_f_{18}$	46.76177	18.06850	52.46139	27.78286	0.004272726
$out1\_f_{19}$	-12.06337	3.22211	0.89449	499.6492	0.03531372
$out1\_f_{20}$	-3.19847	-4.73258	-1.87640	508.2304	0.14344351
$out1\_f_{21}$	12.88944	13.54366	-1.47723	497.2053	0.181095058
$out1\_f_{22}$	-1.21297	-0.77610	-0.44070	502.0313	0.019604057
$out1\_f_{23}$	2.44147	0.11472	0.72269	496.5514	0.079631225
$out1\_f_{24}$	-6.96780	-0.78891	0.40666	494.3632	0.100533104
$out1\_f_{25}$	0.56275	0.09870	-0.17007	497.8733	0.039303016
$out1\_f_{26}$	-0.85591	-0.28260	0.00612	502.0062	0.159647936
$out1\_f_{27}$	0.04226	0.58579	-0.05504	492.7806	0.201552878

After training and developing the new parameters, we determined the different parameters n_i, s_i, t_i, and b_i according to the established rules (Table 3), which represent the coefficients of the final parameters of the model used. To evaluate the evolution of the level of the piezometer I1, the explanatory variable “Level of the reservoir” which is the preponderant variable obtained by reasoning in terms of standard deviation in the HST model, we use as second variable the Season and then the Time (duration of the geotechnical structure). Fig 7-a shows that the season acts sinusoidally on the level of piezometer I1. This reflects and almost constant behavior of the ground. The lower the water level in the dam, the lower the pressure in the ground but a lag may exist between these variations due to the dalay phenomenon. But at certain times, due to the variability of the seasons over time, there are slight differences at the level of the coast of the piezometer I1. This effect proves that the soil reacts well to climatic hazards. The effects of water level and time on the level of this piezometer are

highlighted in Fig 7-b. This influence does not decrease over time, because the two preponderant variables are correlated. Over time, this should decrease due to the consolidation of the soil which should have led to the removal of pore pressures.

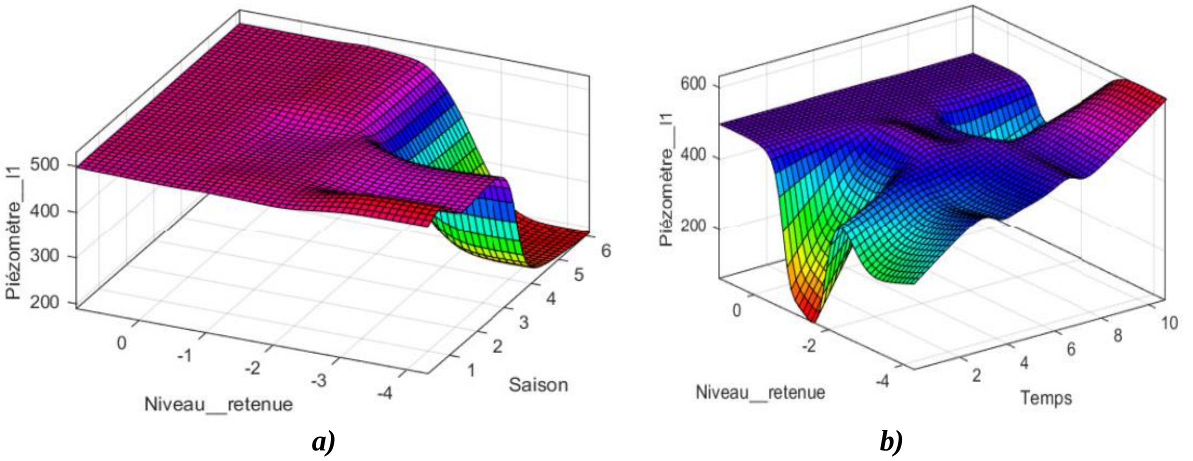

Fig 7. Evolution of the level of piezometer I1 : a) depending on the water level and season ; b) depending on the water level and the weather (lifetime of structure)

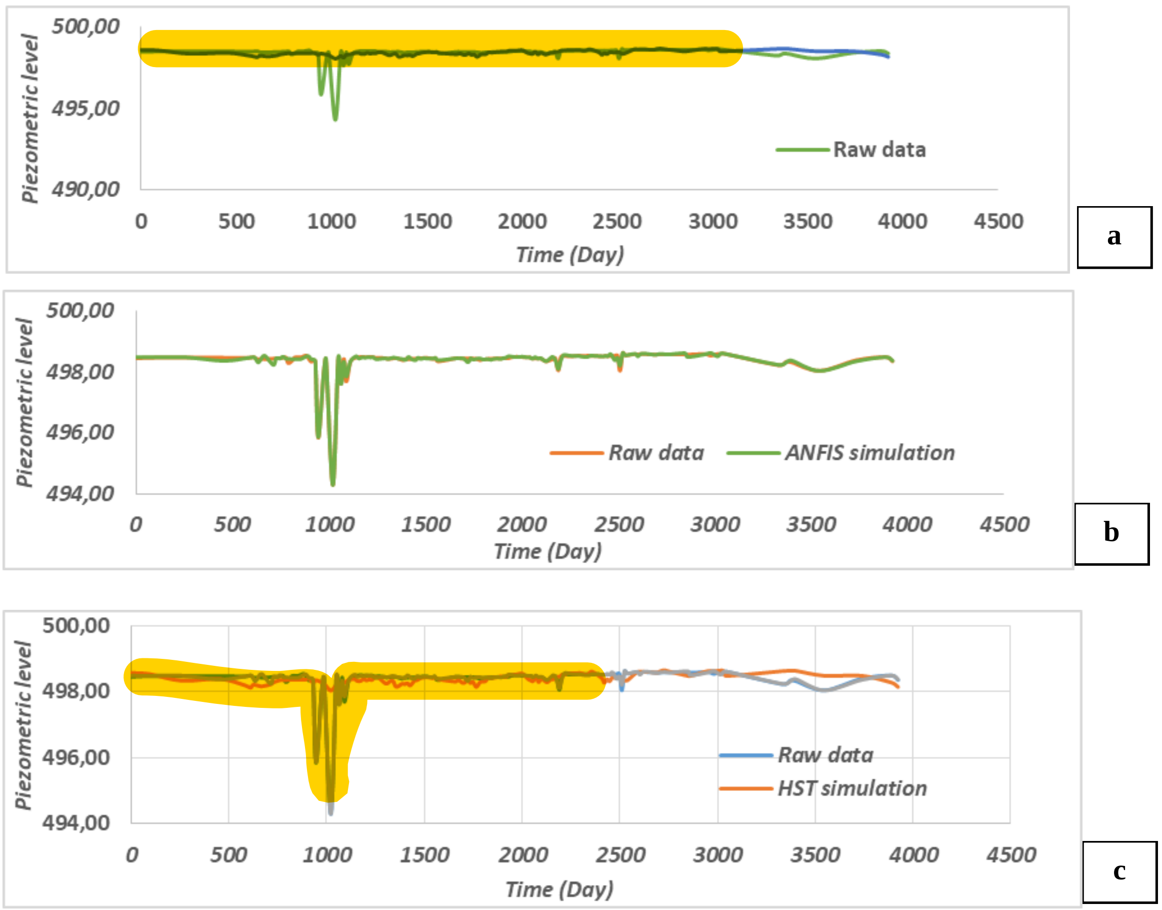

Fig 8. Comparison of the different behavior models of the piezometer I1

Fig 8 indeed shows that the ANFIS model is more compatible with the data observed on the I1 piezometer than the HST model (Fig 8) which is unable to predict the peaks. There is indeed a difference between the two models (Fig 8). It can be seen that the correlation in the case of the ANFIS model is good and centered around a straight line, while in the HST the values are quite scattered (Fig 9). Table 4 presents here the criteria for comparing the different models. It can be seen from Table 4 that the evaluation of the phenomenon based on the fuzzy neuron model describes well the behavior of the piezometric I1, because the mean squared error (MSE) is slow and the correlation coefficient high compared to the HST model.

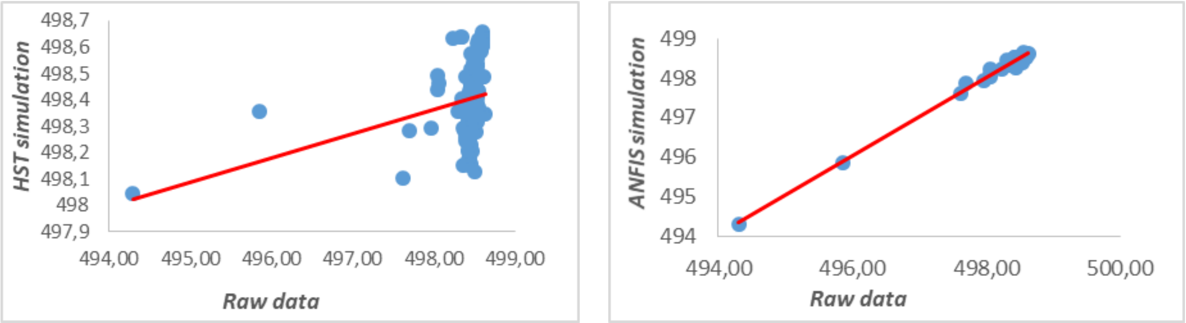

Fig 9. Comparison between the real data and the simulated data of the piezometer I1

This adequacy is justified by the fact that the model makes it possible to highlight the cross-relationships existing between the different explanatory variables. A shortcoming that the conventional model (HST) has due to the assumption of superposition of the said variables. The MAPE criterion (Mean Absolute Percentage Error) i slow, which provesthat the system does not correctly predict the behavior of the piezometer I1 in the short term. D and Dα are parameters of the Kolmogorov-Smirov test.

Table 4. Comparison of HST and ANFIS

	Model	*HST*	*ANFIS*
	MSE	0.18097573	0.00249069
	NASH	-8.8586626	0.98734384
	R	9.21%	98.75%
	MAPE	0.04491726	0.00829865
Kolmogorov-Smirnov	D	0.00015226	0.00018488
	Dα	0.11837281	0.11837281

Assumptions of the Kolmogorov-Smirov test :

- H0 : the simulated values are not significantly different from the real values,
- H1 : the simulated values are significantly different from the real values.

The principle of the test consists in calculating the cumulative distribution of the theoretical proportions and comparing it with those observed. The test statistic is considered to be : D, the maximum difference in absolute value between the observed cumulative proportions and the simulated cumulative proportions [43-45].

$$D = max\|PcO - PcT\|$$

For the piezometer I1 (ANFIS model),

$$D = \ 0.00018488$$

The critical value Dα at threshold α = 5% for a sample of size n (n > 35) is given by:

$D_\alpha = \frac{1.36}{\sqrt{n}}$ either $D_\alpha = 0.11837281$

We see D < Dα so the hypothesis H0is accepted. Then the simulated values of the ANFIS model are not significantly different from the real values.The same analysis process is carried out on all the piezometers. The summary in table 5 presents the different criteria for assessing the model as well as the graphic representations of the piezometers used. To better appreciate the efficiency of the fuzzy neuron model, a comparison is made with the HST model corresponding to each model according to the data from the various piezometers of the dam. Upon analysisof the correlation indicators presented in table 5, it clealy appears that the ANFIS model correctly predicts the phenomenon investigated with correlation coefficient R greater than 80% on the data from all the piezometers.

Table 5. Performance evaluation of the different prediction models on all the piezometers

**Piezometer**	***model***		***HST***	***ANFIS***
E1-1	*MSE*		*1.01654506*	*0.0391054*
	NASH		*0.00876777*	*0.98033811*
	R		*50.22%*	*98.09%*
	MAPE		0.21546735	0.03328576
	Kolmogorov-Smirnov	*D*	0.00022238	0.00036481
		Dα	0.11837281	0.11837281
I1	*MSE*		*0.18097573*	*0.00249069*
	NASH		*-8.8586626*	*0.98734384*
	R		*9.21%*	*98.75%*
	MAPE		0.04491726	0.00829865
	Kolmogorov-Smirnov	*D*	0.00015226	0.00018488
		Dα	0.11837281	0.11837281
I2	*MSE*		*0.7199511*	*0.00722191*
	NASH		*-1.9900762*	*0.99242235*
	R		*25.06%*	*99.25%*
	MAPE		0.1877615	0.01529493
	Kolmogorov-Smirnov	*D*	1.60E-04	3.39E-04
		Dα	0.11837281	0.11837281
I3	*MSE*		*0.08864675*	*0.00649858*
	NASH		*-5.6616545*	*0.93187339*
	R		*13.05%*	*93.63%*
	MAPE		0.03171359	0.00885452
	Kolmogorov-Smirnov	*D*	0.00013798	0.00015548

Piezometer	model		HST	ANFIS
		Dα	0.11837281	0.11837281
I8-2L	*MSE*		*0.00873718*	*0.0031229*
	NASH		*-0.1914663*	*0.7567704*
	R		*45.63%*	*80.57%*
	MAPE		0.01801691	0.0087981
	Kolmogorov-Smirnov	*D*	1.35E-04	1.34E-04
		Dα	0.11837281	0.1183728
M1-1	*MSE*		*0.26905319*	*0.0022615*
	NASH		*-8.8586626*	*0.9873438*
	R		*8.87%*	*99.23%*
	MAPE		0.05758224	0.0081616
	Kolmogorov-Smirnov	*D*	0.00013036	0.0001492
		Dα	0.11837281	0.1183728
I4	*MSE*		*0.06852541*	*0.00252257*
	NASH		*-3.2512176*	*0.96926997*
	R		*19.04%*	*97.02%*
	MAPE		0.03758204	0.00821245
	Kolmogorov-Smirnov	*D*	5.47E-05	6.18E-05
		Dα	0.11837281	0.11837281
I5-2L	*MSE*		*6.9733032*	*0.00297777*
	NASH		*-8.9315753*	*0.99961191*
	R		*9.15%*	*99.96%*
	MAPE		0.31130267	0.01013079
	Kolmogorov-Smirnov	*D*	0.00048475	0.00066052
		Dα	0.11837281	0.11837281
I6-2L	*MSE*		*0.01470121*	*0.00230562*
	NASH		*-5.6992833*	*0.84326898*
	R		*12.99%*	*86.35%*
	MAPE		0.02440705	0.00847497
	Kolmogorov-Smirnov	*D*	7.44E-05	9.49E-05
		Dα	0.11837281	0.11837281
I7-2L	*MSE*		*0.030974*	*0.00658386*
	NASH		*-5.6992833*	*0.84326898*
	R		*27.58%*	*84.61%*
	MAPE		-1.62570427	0.81736629
	Kolmogorov-Smirnov	*D*	1.44E-04	1.55E-04
		Dα	0.11837281	0.11837281
M6-2	*MSE*		*0.04371014*	*0.00889755*
	NASH		*-7.0579013*	*0.78665187*
	R		*11.04%*	*81.89%*
	MAPE		0.02065605	0.01549152
	Kolmogorov-Smirnov	*D*	1.27E-04	1.39E-04
		Dα	0.11837281	0.11837281
M7-2	*MSE*		*0.01880582*	*0.00463144*
	NASH		*-0.595434*	*0.82019766*
	R		*38.53%*	*84.86%*
	MAPE		0.02345939	0.0096185
	Kolmogorov-Smirnov	*D*	1.56E-04	1.52E-04
		Dα	0.11837281	0.11837281
M2-1	*MSE*		*0.7199511*	*0.00722191*
	NASH		*-1.9900762*	*0.99242235*
	R		*25.06%*	*99.25%*
	MAPE		0.1877615	0.01529493
	Kolmogorov-Smirnov	*D*	1.60E-04	3.39E-04
		Dα	0.11837281	0.11837281
M3-1	*MSE*		*0.2891059*	*0.0034373*
	NASH		*-3.2128611*	*0.99029053*
	R		*19.18%*	*99.04%*
	MAPE		0.08921857	0.0097318
	Kolmogorov-Smirnov	*D*	1.13E-04	1.60E-04
		Dα	0.11837281	0.11837281
M4-1	*MSE*		*0.02583915*	*0.00258702*
	NASH		*-0.4467653*	*0.9368043*
	R		*40.87%*	*94.08%*
	MAPE		0.0346867	0.00819494
	Kolmogorov-Smirnov	*D*	8.47E-05	8.69E-05
		Dα	0.11837281	0.11837281
M5-1	*MSE*		*0.05308272*	*0.00940327*

Piezometer	*model*		*HST*	*ANFIS*
	NASH		*0.5238724*	*0.93941527*
	R		*67.74%*	*94.29%*
	MAPE		0.0367271	0.01289637
	Kolmogorov-Smirnov	*D*	0.00032319	0.00030788
		Dα	0.11837281	0.11837281
	MSE		*0.03553419*	*0.00063743*
	NASH		*-0.4264033*	*0.98932728*
M5-2	*R*		*41.21%*	*98.95%*
	MAPE		0.21546735	0.03328576
	Kolmogorov-Smirnov	*D*	0.00022238	0.00036481
		Dα	0.11837281	0.11837281
	MSE		*0.01880582*	*0.00463144*
	NASH		*-0.595434*	*0.82019766*
M8-2	*R*		*38.53%*	*84.86%*
	MAPE		0.02345939	0.0096185
	Kolmogorov-Smirnov	*D*	1.56E-04	1.52E-04
		Dα	0.11837281	0.11837281
	MSE		*0.00460024*	*0.01644953*
	NASH		*-0.118744*	*0.82498519*
M8-1	*R*		*47.20%*	*85.23%*
	MAPE		0.02308931	0.01074947
	Kolmogorov-Smirnov	*D*	1.48E-04	1.34E-04
		Dα	0.11837281	0.11837281
	MSE		*0.97957829*	*0.17354932*
	NASH		*-0.0800774*	*0.89717252*
M9-1	*R*		*48.08%*	*90.80%*
	MAPE		0.19712565	0.0621059
	Kolmogorov-Smirnov	*D*	1.67E-04	2.22E-04
		Dα	0.11837281	0.11837281
	MSE		*1.60972866*	*0.05971698*
	NASH		*0.00337126*	*0.9809826*
M10-1	*R*		*50.08%*	*98.15%*
	MAPE		0.26691034	0.04057676
	Kolmogorov-Smirnov	*D*	3.26E-04	4.17E-04
		Dα	0.11837281	0.11837281
	MSE		*0.88717832*	*0.03048639*
	NASH		*-0.0773726*	*0.98172326*
M10-2	*R*		*48.14%*	*98.22%*
	MAPE		0.19400747	0.02962152
	Kolmogorov-Smirnov	*D*	2.83E-04	4.20E-04
		Dα	0.11837281	0.11837281

4.1.2 Piezometer M1-1

The basic model (HST) tends to generalize the system, and approximates the behavior of the piezometer more or less well until 2015 when the peaks become larger and larger, from then on the HST model does not can’t keep up anymore. There are then notable differences between the two models. Fig 10-b shows us the effect of water level and time on the level of piezometer M1-1. It can be seen that the water level in the dam significantly influences the piezometry. Over time, this should decrease due to the consolidation of the soil, which should have led to the removal of the voids. It is noted in Fig 10-a, that the season acts in a sinusoidal way on the coast of the piezometer M1-1. This reflects an almost constant behavior of the ground. But at certain times, due to the variability of the seasons over time, there are slight differences at the level of the M1-1 piezometer level. This proves that the soil reacts well to

climatic hazards. It is also interesting to note thatin neighborhoods of the NR (Normal Reservoir), the piezometric level does not change almost whatever the season, this shows the preponderance of the influence once again of the level of the reservoir with respect to the season.

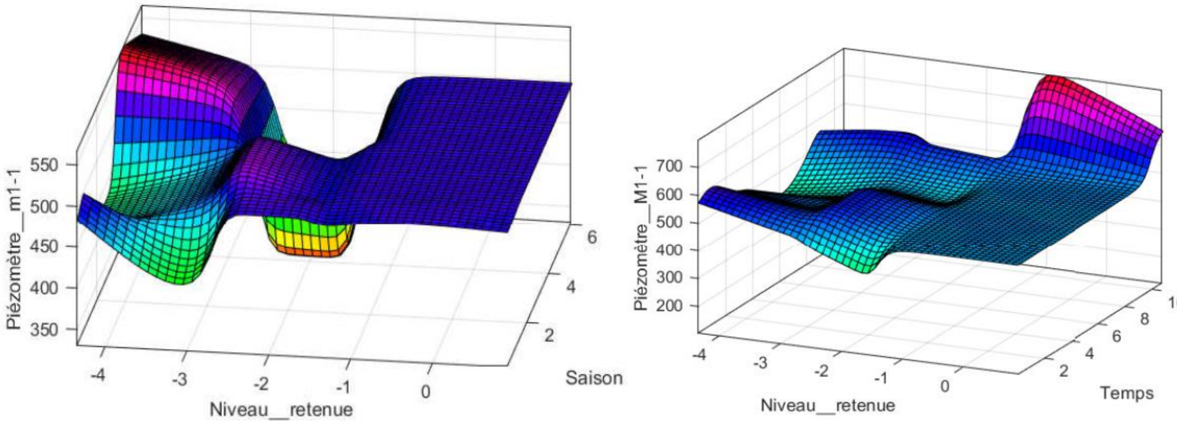

a) *b)*

Fig 10. Evolution of the piezometer level: a) M1-1 as a function of the water level and the season; b) M1-1 depending on the water level and time- M1-1 depending on the water level and time (lifetime of the structure)

4.1.3. Piezometer I2

Fig 11-a shows us the influence of the level of the reservoir and time on the levelo f the piezometer I2, we notice immediately that as for the previous piezometers, the level of the reservoir has a significant influence on the piezometric level, in fact, we notice that around 0 the piezometric level hardly changes regardless of the time, yet logic would dictate that the consolidation of the ground over time also leads to a decrease in the influence of the level over time.

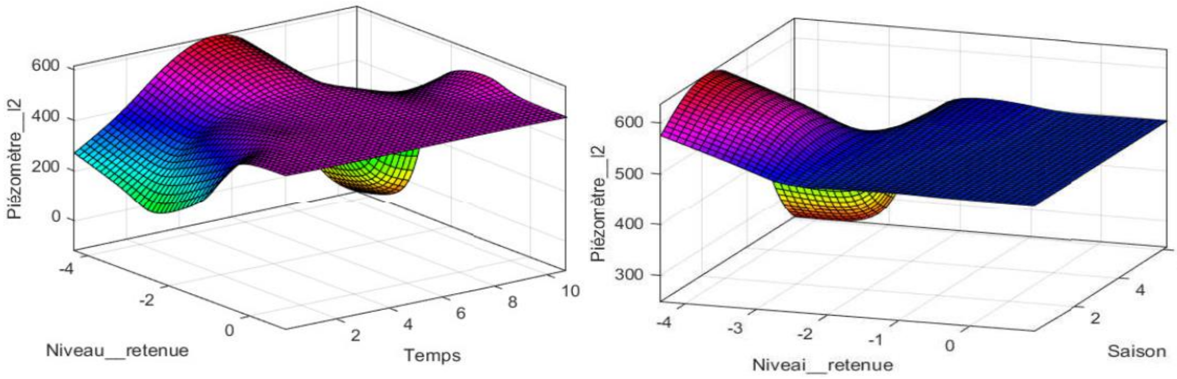

a) **b)**

Fig 11. Evolution of the piezometer I2 level : a) Depending on the water level and time ; b) According to the coast of the water and the season

Fig 11-b shows us the influence of the level of the reservoir and the season on the level of the piezometer I2, we notice immediately that as for the previous piezometers, that the season remains mute compared to the level of the reservoir at the around the normal retention level, thus the level of the reservoir once again demonstrates the preponderance of its influence whatever the season, even ignoring the contraction and expansion of the concrete.

4.1.4. M3-1 piezometer

The ANFIS model is more compatible with the data observed on the M3-1 piezometer, indeed it manages to predict all the variations and follows the peaks very well unlike the HST model which has great difficulty in predicting the behavior of the piezometer at the level of sudden variations. One notices again the domination of the influence of the piezometry in the neighborhoods of the normal reservoir, the weather hardly influences the level of the piezometry when the level of the reservoir is normal, which is almost always the case. We can underline the constant character whatever the season of the piezometric level in the vicinity of 0 which represents the average of the levels of reservoir, which shows the strong influence of the level of the reservoir compared to the season on the behavior of the piezometer M3-1 (Fig 12).

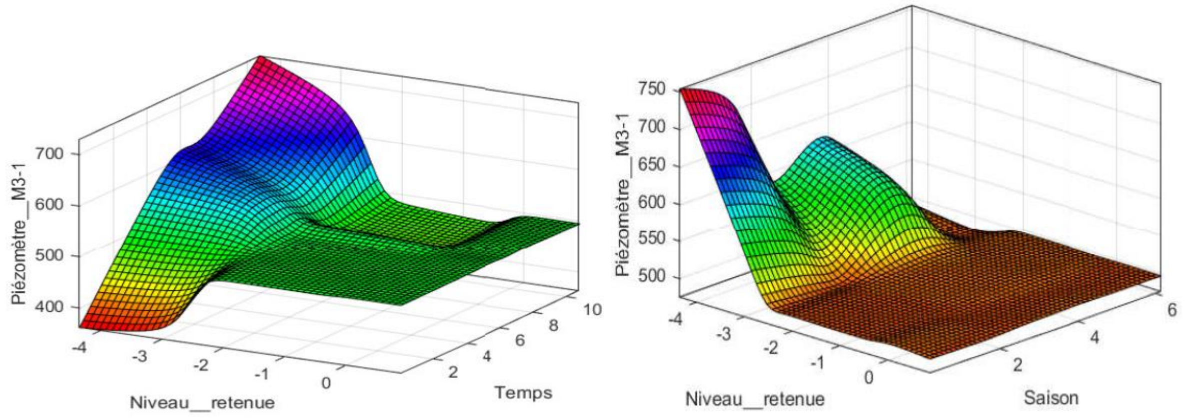

a) b)

Fig 12. Evolution of the elevation of the M3-1 piezometer: a) Depending on the water level and time (lifetime of the structure); b) According to the coast of the water and the season

4.1.5 Piezometer I3

One can immediately appreciate the power of the ANFIS model which apprehends the behavior of the piezometer very well and which faithfully predicts the results of the piezometer I3 compared to the HST model which always has a lot of difficulty to predict peaks, even if the prediction is acceptable. It is important to emphasize, apart from the strong influence of the reservoir level, that the piezometric levels remain much higher upstream than downstream, which testifies to the effectiveness of the scaling layer and the non-communication of water between the two galleries. The piezometric levels downstream remain lower than the levels upstream regardless of the season, which testifies to the effectiveness of the scaling layer. Note also here the strong influence of the level of the reservoir (Fig 13).

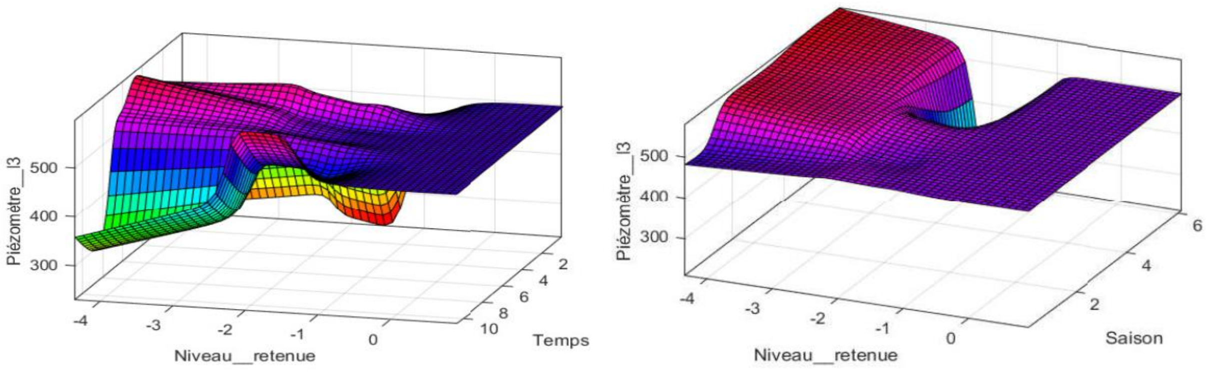

a) **b)**

Fig 13. Evolution of the elevation of the piezometer I3: a) as a function of the elevation of the water level and time (lifetime of the structure); b) depending to the water coast and the season

4.1.6. Piezometer M8-1

Fig 14 shows the evolution of the water level in this piezometer as a fuction of time. The ANFIS model is more compatible with the data observed on the M8-1 piezometer, indeed it menages to predict all the variations and follows the peaks very well unlike the HST model which great difficulty in predicting the behavior of the piezometer at the level of sudden variations.

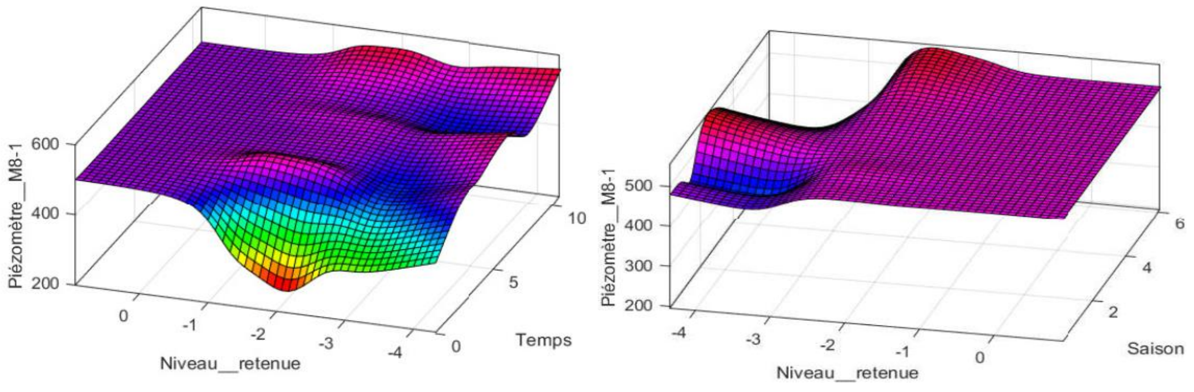

a) **b)**

Fig 14. Evolution of the elevation of the piezometer M8-1: a) depending on the elevation of the water and time (lifetime of the structure); b) according to the elevation of the water and the season

4.1.7. Piezometer I8-2L

Fig 15-a shows us the influences of the reservoir level and time on the I8-2L piezometer level, as for all the other piezometers of the intake dam, the reservoir level has significant influence on the piezometer level which hardly varies around 0. Fig 15-b shows the influences of the level of the reservoir and the season on the piezometer level I8-2L, as for all the other piezometer level which hardly varies around 0.

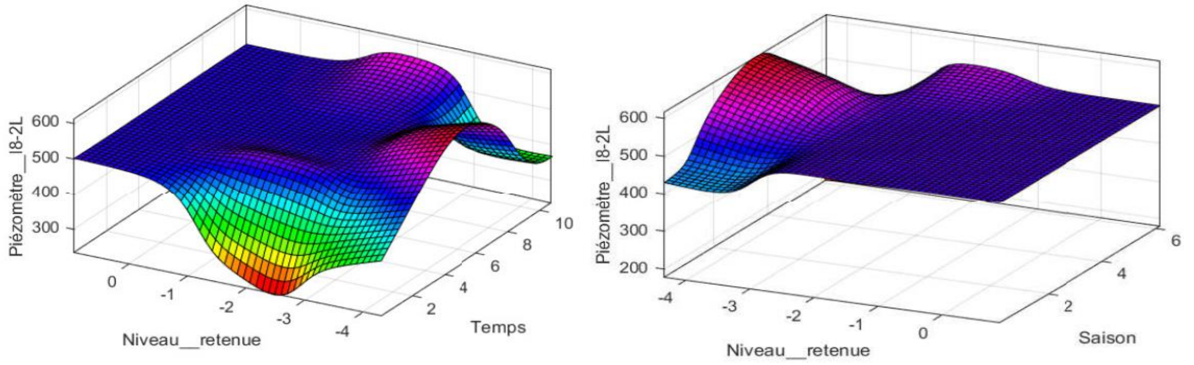

a) b)

Fig 15. Evolution of the elevation of the piezometer I8-2L: a) depending on the elevation of the water and time (lifetime of the structure); b) according to the elevation of the water and the season

4.1.8. Piezometer M9-1

I can be seen that the two models apprehend the behavior of the M9-1 piezometer quite well when it operates in an almost linear manner (Fig 16-c). But the various observable peaks are

well estimated by the ANFIS model (Fig 16-b) while the basic HST model (Fig 16-a) generalizes the behavior of the piezometry. Fig 17-a, shows the influence of the level of the reservoir and of time on the piezometer level M9-1, unlike the other piezometers of the intake dam, the influence of the level of the reservoir drops significantly and a remarkable increase is observed the influence of time, with a piezometric level which incrases with time. Fig 17-b shows the influence of the reservoir level and the season on the M9-1 piezometer level, unlike the other piezometers of the intake dam, the influence of the reservoir level drops significantly and an increase remarkable the influence of the season through slight oscillations around the level of the normal reservoir.

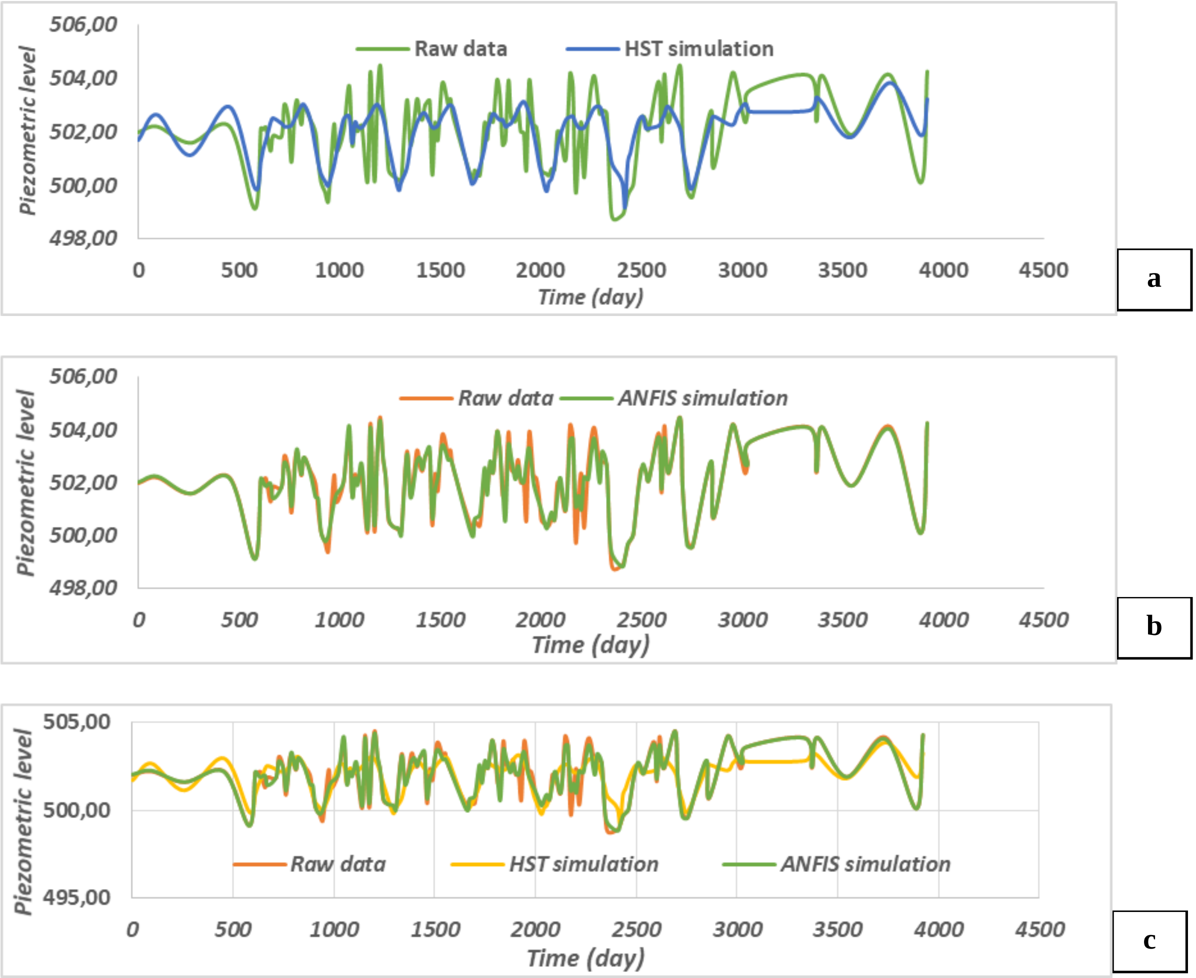

Fig 16. Comparison of the different behavior models of the M9-1 piezometer

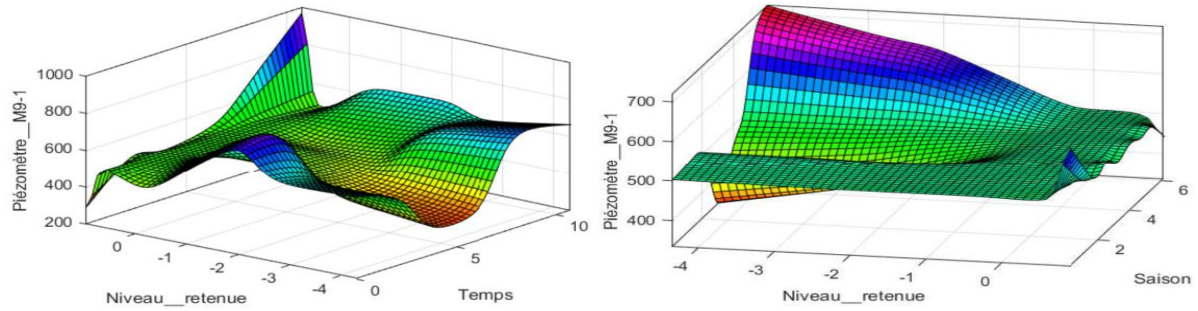

a) **b)**

Fig 17. Evolution of the elevation of the piezometer M9-1: a) depending on the coast of the water and the weather; b) according to the coast of the water and the season

4.1.9. Piezometer M10-1

It can be seen that the two models apprehend quite well the behavior of the M10-1 piezometer when it operates in an almost linear maner. The Fig. 18-a, highlights the influences of the level of the reservoir and of time on the level piezometer M10-1. The influence of the level of the reservoir decreases significantly and one observes a remarkable increase in the influence of the time better although on the M10-1 piezometer, whit a piezometric level which increases with time, should we conclude thaht it is linked to the fact we are approaching the spillways dam ? Fig 18-b shows us the influences of the level of the reservoir and the season on the piezometer level M10-1, the influence of the level of the reservoir drops significantly and we observe a remarkable increase in the influence of the season through slight oscillations around the normal restraint level. The behavior of the dam through all piezometers is represented in table 5. This behavior is typical on the ground of this region [46].

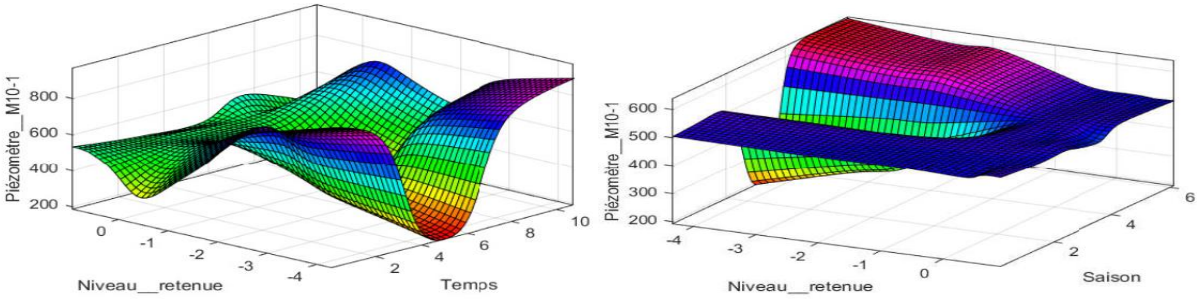

a ***b)***

Fig 18. Evolution of the M10-1piezometer elevation : a) as a function of the water level and the weather (lifespan of the structure) ; according to the elevation of the water and the season

4.2. Spillway dam : piezometric data analysis

Fig 19 shows the influences of the level of the reservoir and of the season on the piezometer level E1-1. The influence of the level of the reservoir decreases significantly and we observe a

remarkable increase in the influence of the season through oscillations around the normal restraint level. The results presented in this part for the analysis of the subpressures show that the water level in the dam significantly influences the piezometry at the level of the intake dam, we note at the level of this same dam that when we are at the normal reservoir level, the piezometric level hardly changes whatever the season or the whaterver, apriori this observation is worrying since the consolidation of the groung should logically reduce the influence of the level of the reservoir over time, but the lack of data from past periods and on the geology of the soil makes any conclusion premature ; this beings the case, it is nonetheless certain that the height of the reservoir has a preponderant influence on the piezometric level of the intake dam. From plot 9 (in the vicinity of the spillway dam) there is a significant increase in the influences of the season and the weather and a significant decrease in the influence of the level of the reservoir on the piezometry. This analysis remains valid throughout. Along the spillway dam, these fluctuations can be explained by the variations in activity on this dam according to the seasons (low water level, indeed during these periods, the spillways are open to evacuate the excess water in the dam, and are closed during periods of low flood), this dam is also the most affected by the Alkali-Granulate-Reaction, which helps to observe strong seasonal variations.

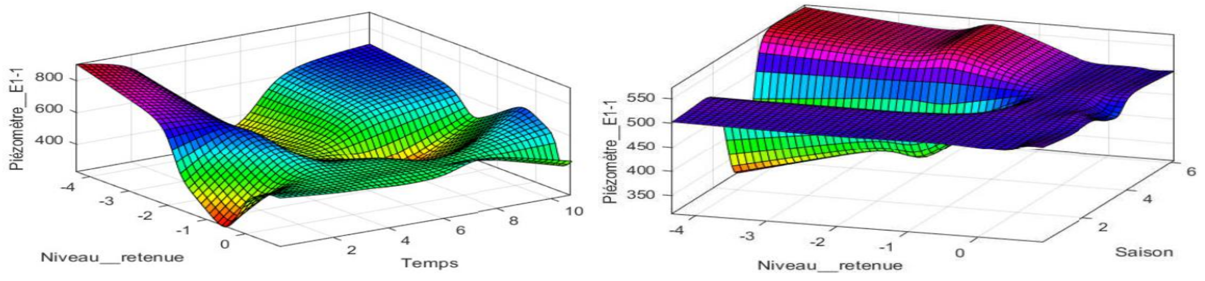

Fig 19. Evolution of the elevation of the E1-1 piezometer as a function of the water elevation and time and depending on the water elevation and the season

4.3 Displacement measurements from 1997 to 2019

4.3.1 Displacements in the pendulum P1D

After applying the HST and ANFIS models to the raw data of the P1D pendulum, the results presented through Fig 20 were obtained. Overall, the behavior of the pendulum is well assimilated by the models, and as shown in the figures above, the errors are acceptable ; nevertheless, the excellence of the model is mainly explained by the quality of the influences of the explanatory data which are the level of the reservoir, the season in the year and the time

(HST). The statistical analysis of the influential parameters recorded in this pendulum is op good quality (correlation coefficient R^2 =0.99412 for Upstream-Downstream and R^2 = 0.97767 for left-right). It shows the low influence of the season on the value of the measurements (4.949 % contribution to the explanation of the phenomena for Upstream-Downstream and 17.236% for left-right- «Seasonal effects» graphs). The analysis showsan upward temporal drift, far too upward moreover, of the variations recordedon the pendulum (95.151% explanation for Upstream-Downstream and 92.095% for left-right) resulting in large movements unstabilized 7.44 cm for Upstream-Downstream and 7.11 cm for left-right in 20 years (« Time effects » graphs). A weakly explanatory hydrostatic effect (2.36% for Upstream-Downstream and 2.996 % for left-right) resulting in a displacement of 0.39 cm for a rise in the water level of 1 m Upstream-Downstream and by a displacement of 0.1 cm for a rise in the level of the water body of 1 m in left-right bank (see « Hydrostatic Effects » graphs).

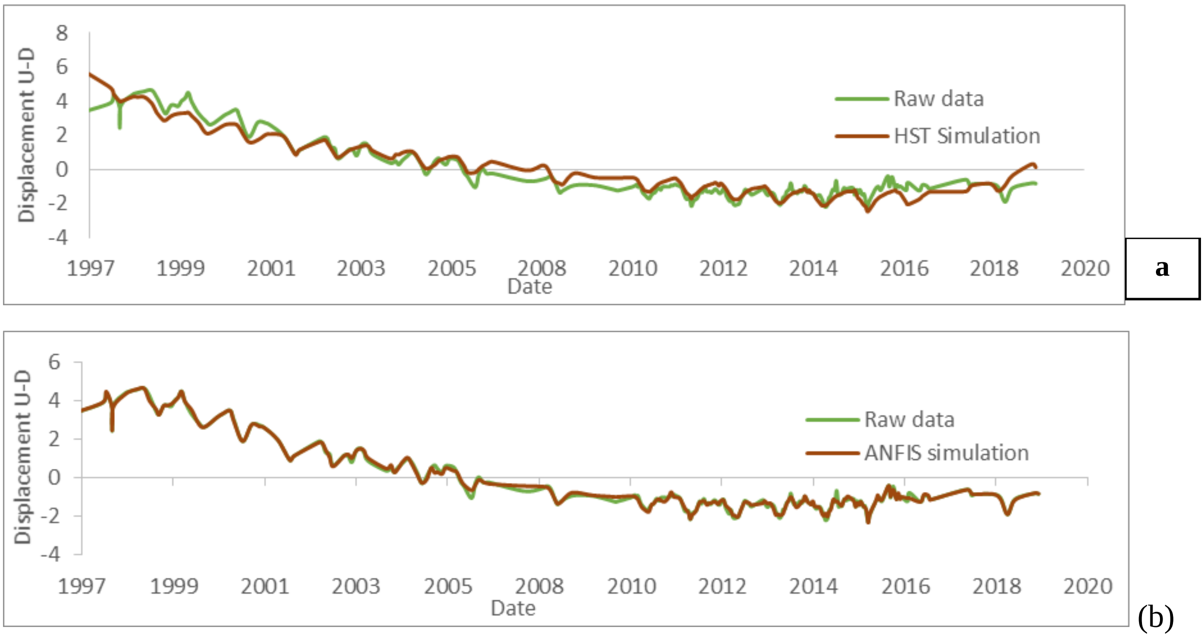

Fig 20. Displacements from upstream to donwstream measured (cm) on the pendulum P1D ; a) HST, b) ANFIS

4.3.2 Displacements in the pendulum P3I

Fig 21 highlight the evolution of displacements in the dam according to the years of service. The influence of the explanatory data of all the pendulums is illustrated through table 6.

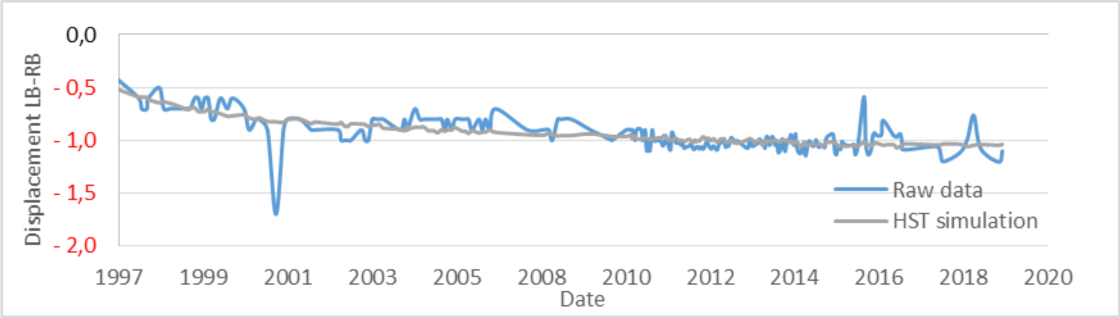

b)

a

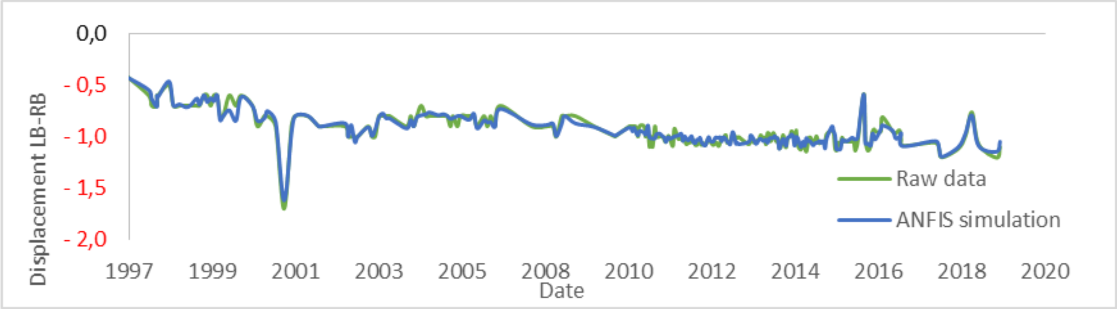

b

Fig 21. Displacement (cm) Left-Right bank of the pendulum P3I : a) HST ; b) ANFIS

Table 6. Evaluation of the performances of the different prediction models on all the pendulums

Pendulum	Model	HST	ANFIS
E6-7D	MSE	0.26480258	1.28E-05
	NASH	0.81610649	0.99999248
	R	0.84467056	0.99999248
	MAPE	-3.72081555	-0.02277325
E2-3D	MSE	0.0587672	1.47E-06
	NASH	0.99864049	0.99999997
	R	0.99864234	0.99999997
	MAPE	-0.51834962	-0.00160254
P5I	MSE	0.03667014	1.29E-08
	NASH	0.92078521	0.99999997
	R	0.9265996	0.99999997
	MAPE	2.16593849	0.00104804
P3I	MSE	0.02367039	0.00659559
	NASH	0.98203608	0.99505795
	R	0.98235309	0.99508281
	MAPE	2.46122631	1.20555695
P1D	MSE	0.41384878	0.01361954
	NASH	0.85262718	0.99575404
	R	0.87155629	0.99577299
	MAPE	-62.5843528	-3.36682009

4.4 Relocation of the spillway dam

4.4.1. Pendulum E2-3D

Fig 22 and 23 show the evolution of the displacements recorded in this pendulum over the years. Table 6 summarize the influences of the reservoir coast, season and time in the E2-3D pendulum.

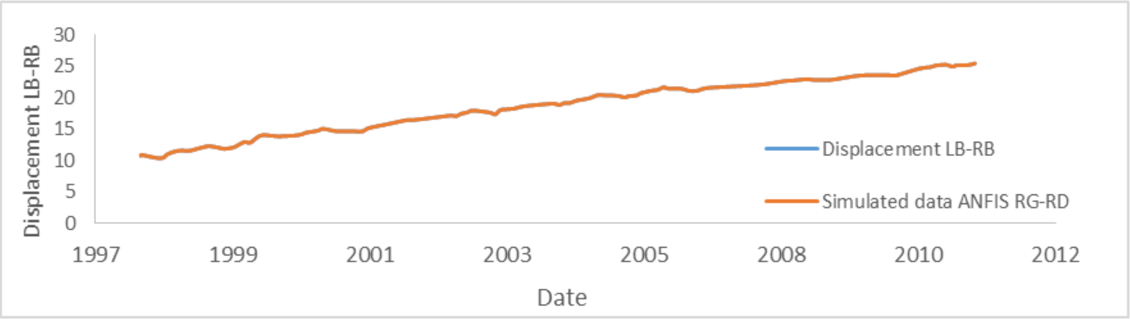

Fig 22. Displacement (cm) of the Left-Right bank of the pendulum E2-3D

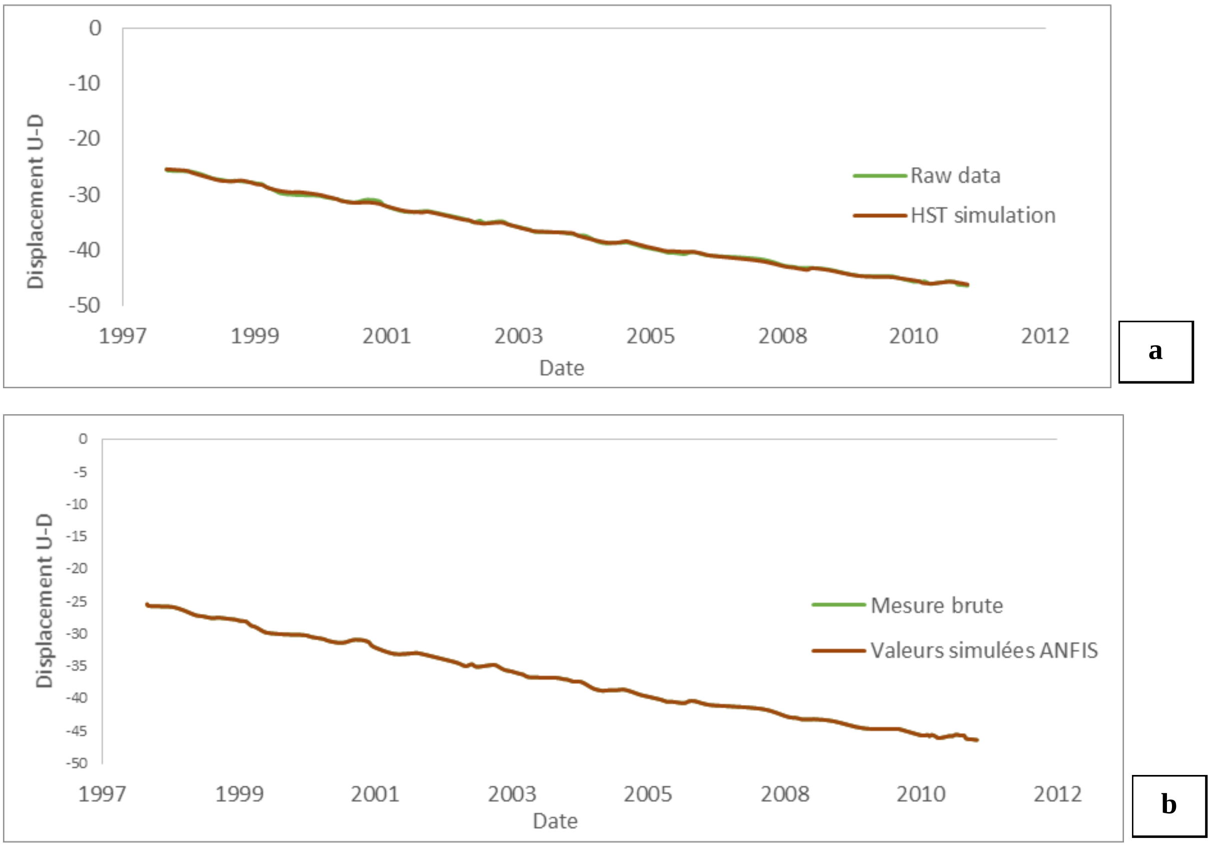

Fig 23. Displacement (cm) upstream-downstream pendulum E2-3D : a) HST ; b) ANFIS

4.4.2. Pendulum E6-7D

Table 6 summarize the influences of the reservoir coast, season and time in the E6-7D pendulum. Fig 24 shows the evolution of displacements as a fuction of time.The analysis of the monitoring data of the pendulums of the large earthen dam of Songloulou gives the results above. These results are presented in the form of graphs for each of the explanatory effects

resulting from the associated statistical analysis. This analysis is goodquality. It shows the low (average) influence of the season on the value of the measurements. The analysis also shows an upward temporal drift, far too upward moreover, of the variations recorded on the pendulum resulting in large unstabilized movements and a weakly explanatory hydrostatic effect for explanation. This very strong influence of time, although abnormal, is logically explained by the phenomena of alkali-aggregate-reaction, which promotes accelerated aging of the structure. The presence of many cracks on the structure could also contributes. From the analysis of the data of the various pendulums, it appears that the displacements on the spillway dam are very important.

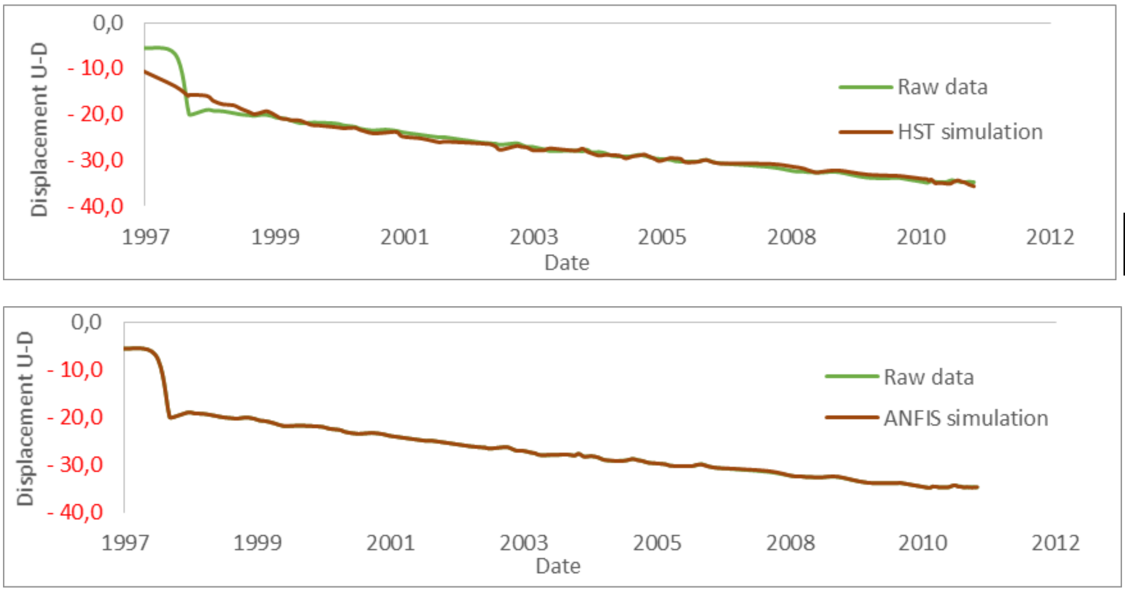

a

b

Fig 24. Upstream-Downstream displacements of pendulum E6-7D : a) HST ; b) ANFIS

This phenomenon is explained by the fact that this dam is affected by the alkali-aggregate-reaction and by the fact of significant stresses during periods of flooding (openings and closings of the spillways). In order to obtain the behavior indicators at any point of this earth dam under different water level, a numerical modeling is carried out from GEOSTUDIO calculation software for analyszed the displacements, the stresses and slope stability.

5. Numerical simulation of the water level of the earthen dam on GEOSTUDIO

GEOSTUDIO is a Finite element calculation code to perform any type of mechanical analysis for various geotechnical structures [47]. The water intake dam is modeled in 2D plane strains representings its cross section. All displacements and seepage or flow condition are fixed at

the bottom of the model. The mechanical behavior of the dam materials obeys of the Mohr-Coulomb failure criterion [48-49]. Table 7 presents the parameters of the different materials of the water intake dam. From the water levels in the reservoir, observed according to the seasons, three piezometric levels were selected for the modeling (minimum : 522 m, normal : 528 m and maximum level : 530 m). In order to appreciate the overall behavior of the structure, the results in stresses, displacements and factor of safety of slope are given in cartographic form on Fig 25 to 36.

Table 7. Parameters of subsoil and earth materials

Material	γ_{unsat} (kN/m^3)	γ_{sat} (kN/m^3)	c'(kPa)	Φ'(°)	Young's modulus (MPa)	Poisson's ratio (-)	Coefficient of permeability (m/s)
Core	18	19	15	32	74	0.3	1.15741E-14
Rip-Rap	22	24	0	40	80	0.3	1.15741E-12
Filtered	19	22	0	40	400	0.3	2.3148E-12
Rockfill	22	24	0	45	800	0.3	1.15741E-12
Rock foundation	26	26	1000	45	5000	0.3	5.78704E-14

At the minimum water level 522 m, the Fig 25 to 28 show the actual behavior of the earthen dam. The greatest displacements are obtained at the top of the structure. Thes shear failure curve of the dam is clearly visible, reflecting the most vulnerable part of the dam subjected to fluctuations in the water level.

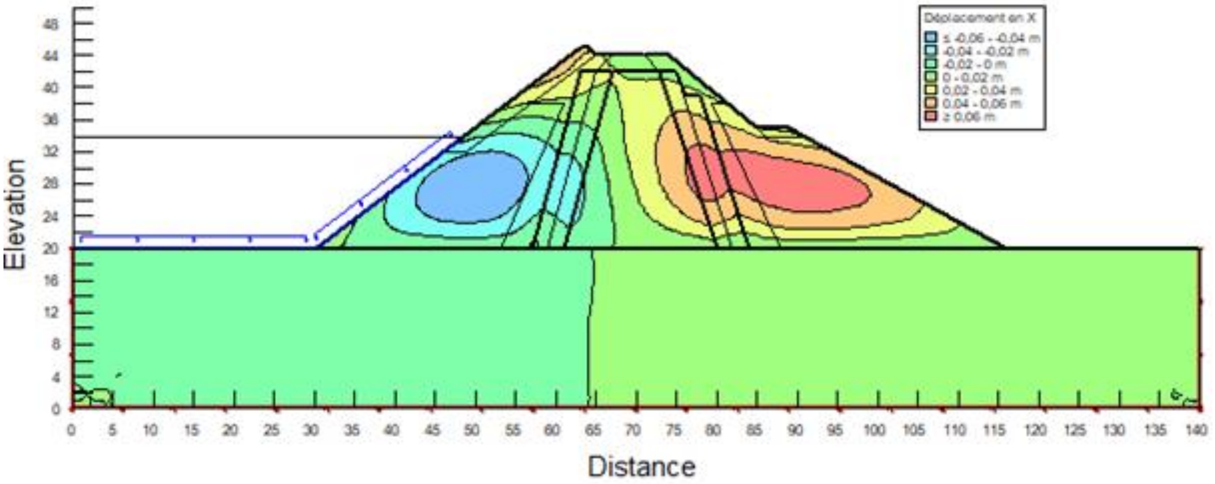

Fig 25. Horizontal displacements at the water level 522 m

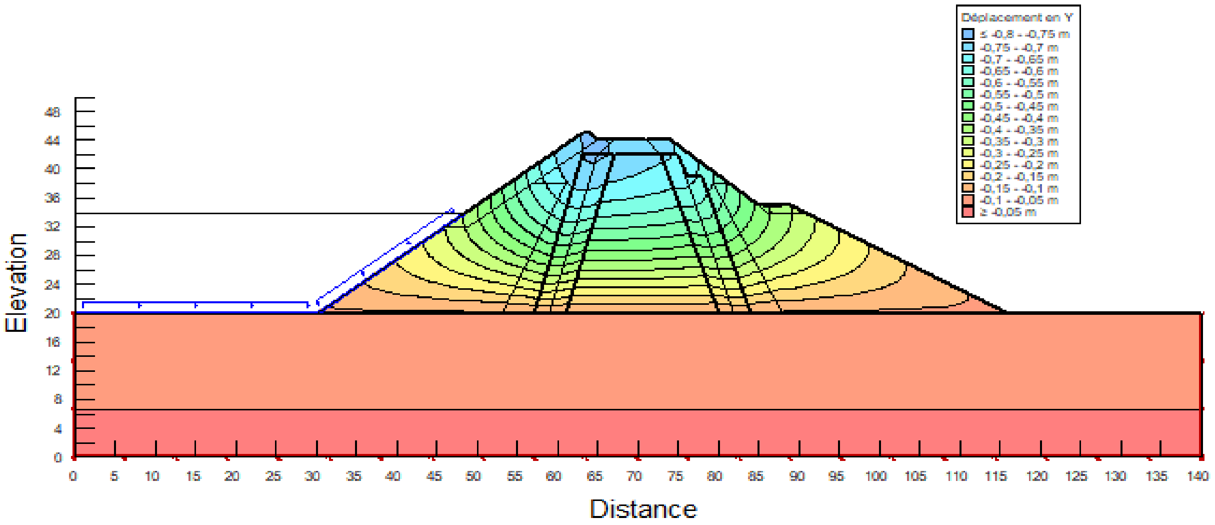

Fig 26. Vertical displacements at the water level 522 m

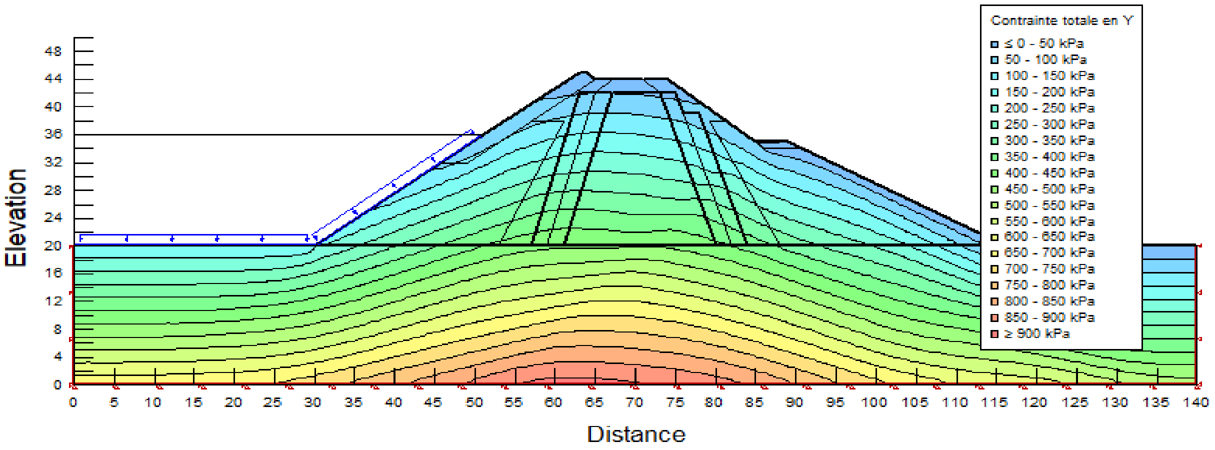

Fig 27. Total stress at the water level 522 m (900 kPa at the bottom of the earth dam)

The maximum horizontal displacement is 6 cm and vertical displacement is 74 cm. The maximum total stress at the minimal water level is located at the bottom of the model, and is 900 kPa.

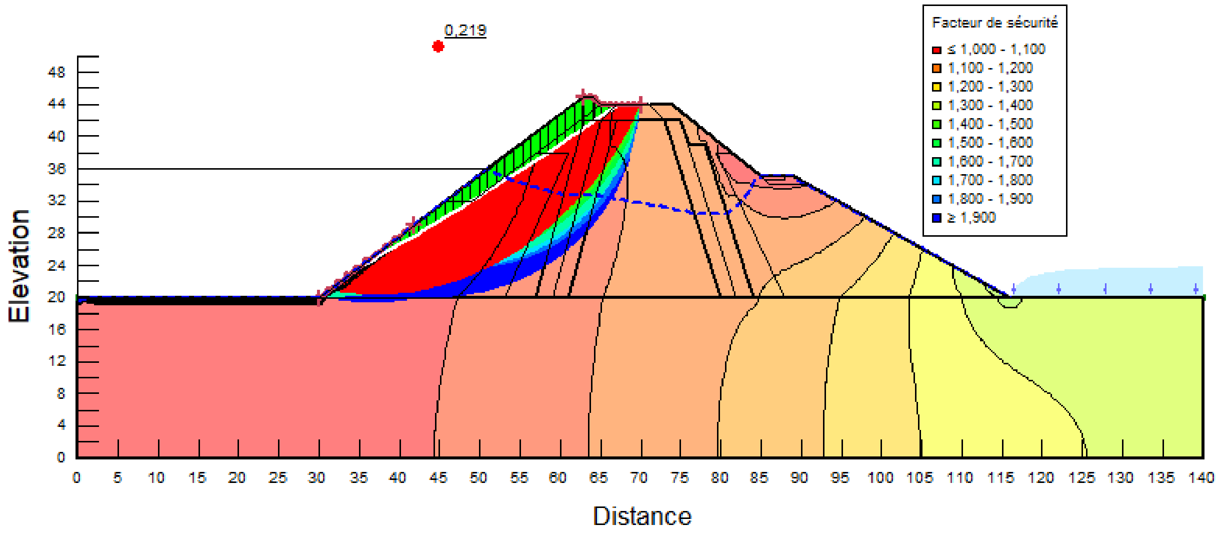

Fig 28. Critical slope stability of earthen dam of Songloulou at the water level 522 m

At the normal water level 528 m, the Fig 29 to 32 show the actual behavior of the earthen dam. The greatest displacements are obtained at the top of the earth dam. The shear failure line of the dam is clearly visible, reflecting the most vulnerable part of the dam subjected to fluctuations in the water level. The maximum horizontal displacement is 11.68 cm and vertical displacement is 80 cm.

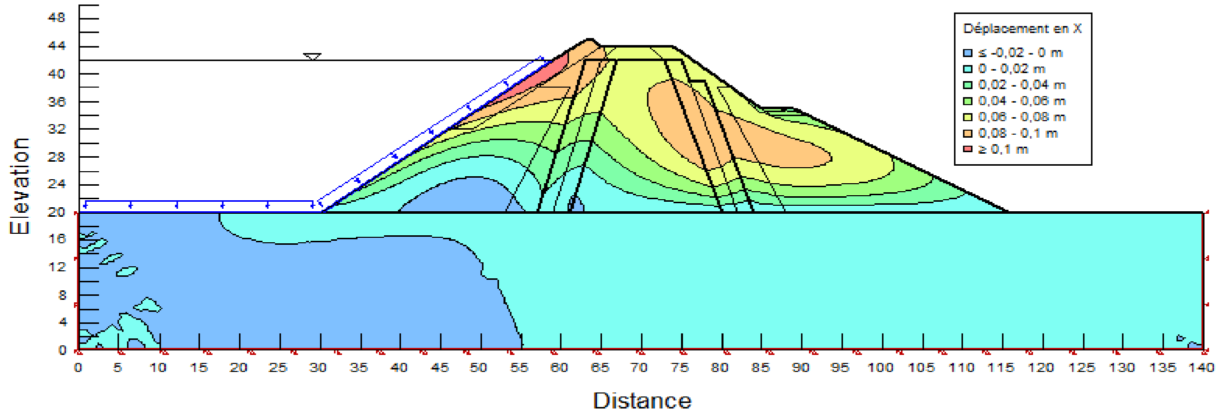

Fig 29. Horizontal displacements at the water level 528 m

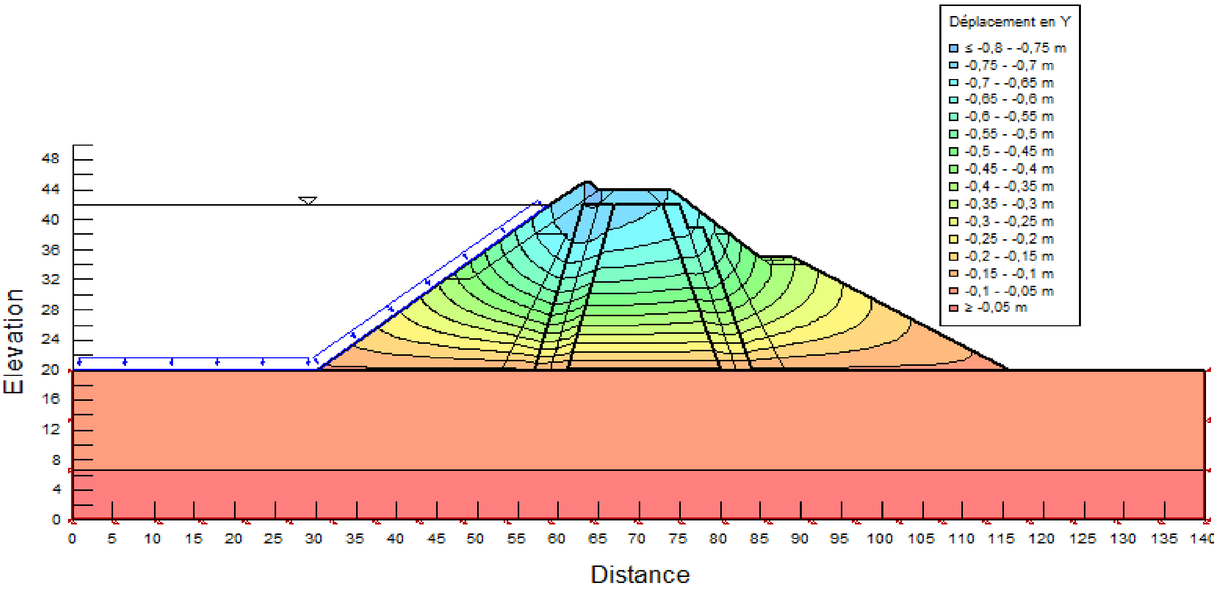

Fig 30. Vertical displacements at the water level 528 m

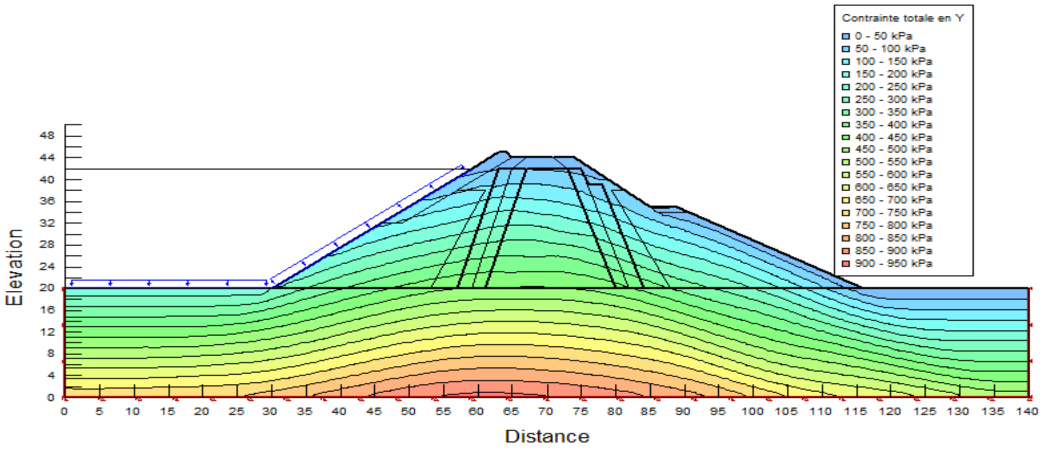

Fig 31. Total stress (912 kPa at the bottom of the earth dam) at the water level 528 m

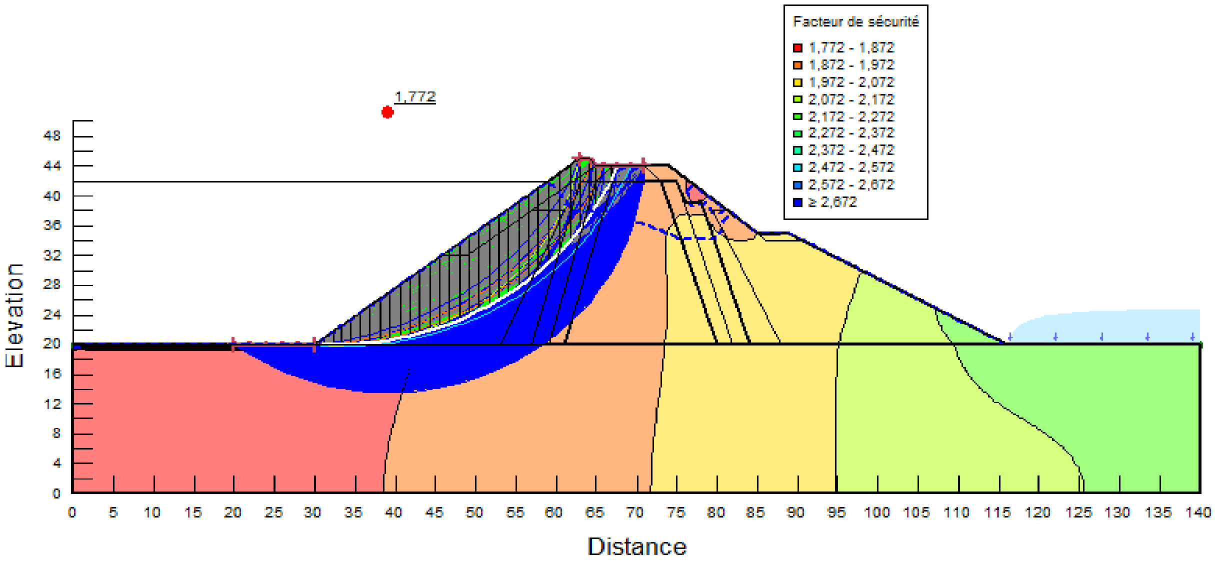

Fig 32. Critical slope stability of earthen dam of Songloulou at the water level 528 m

The maximum total stress at the minimal water level is located at the bottom of the model, and is 912 kPa. At the maximal water level 530 m, the Fig 33 to 36 show the actual behavior of the earthen dam. The greatest displacements are obtained at the top of the earth dam. The shear failure line of the dam is clearly visible, reflecting the most vulnerable part of the dam subjected to fluctuations in the water level. The maximum horizontal displacement is 18 cm and vertical displacement is 80 cm. The maximum total stress at the minimal water level is located at the bottom of the model, and is 920 kPa. Through these modeling, the results of global displacement according to the level of the reservoir of the dam show that it remains stable as a whole. All these displacements are less than 20 cm. In an advanced monitoring process, we used the auscultation data to have a realistic modeling.

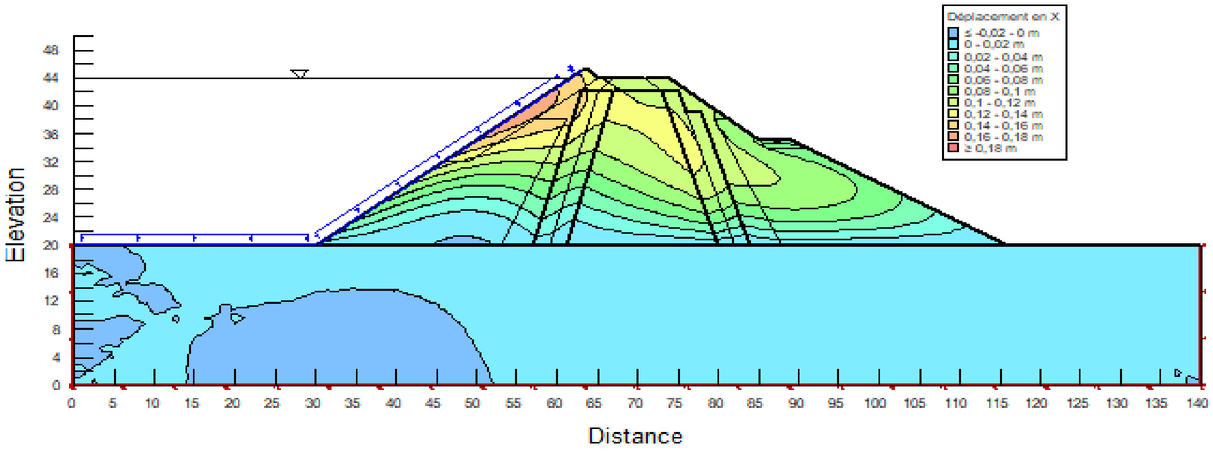

Fig 33. Horizontal displacements at the water level 530 m

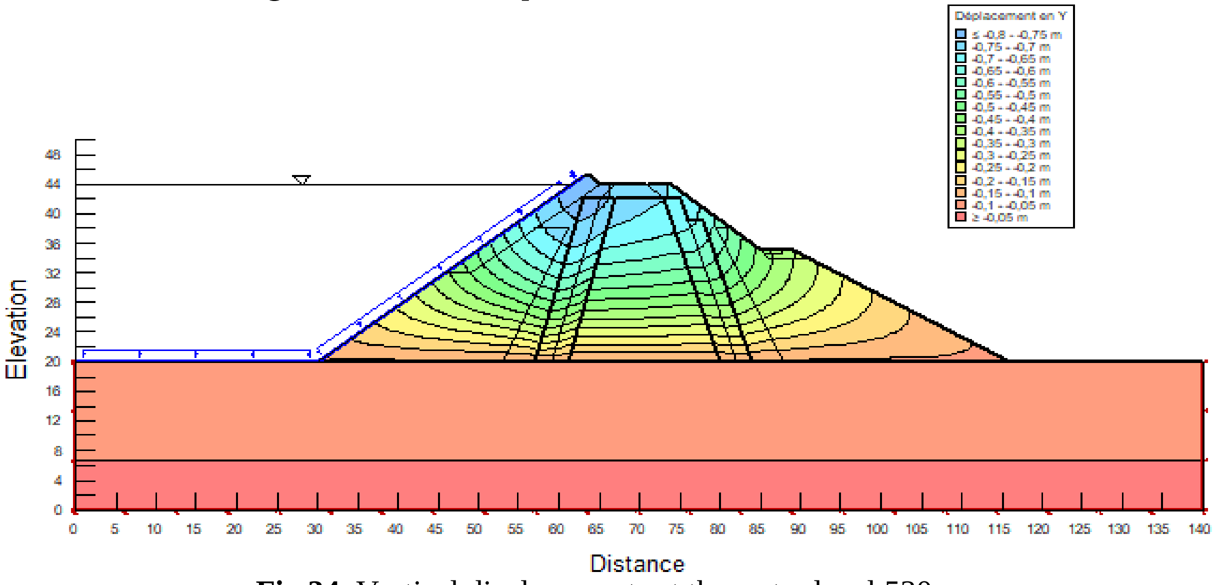

Fig 34. Vertical displacements at the water level 530 m

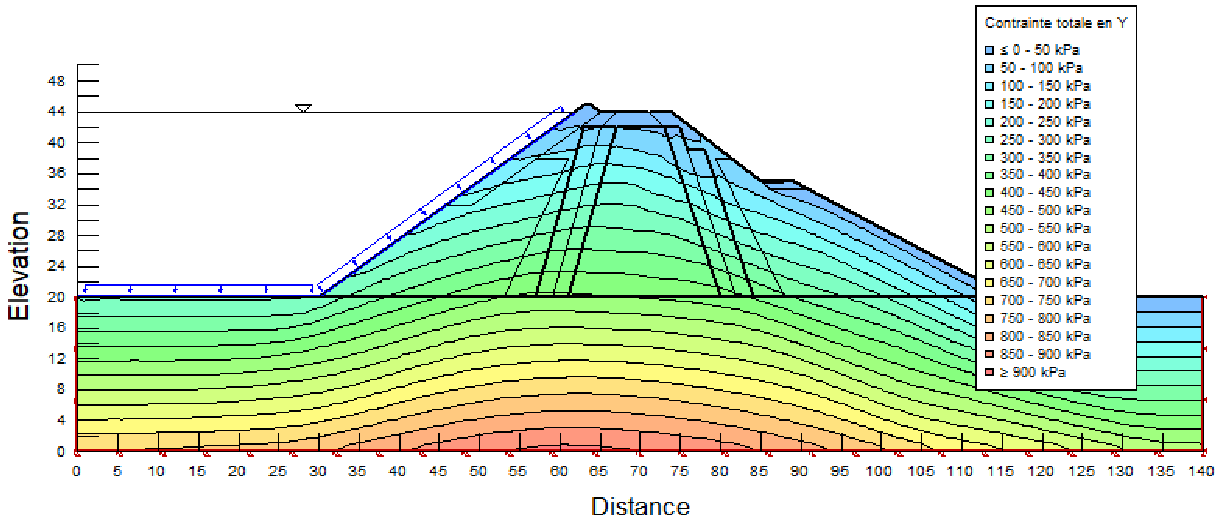

Fig 35. Total stress at the water level 530 m (920 kPa at the bottom of the earth dam)

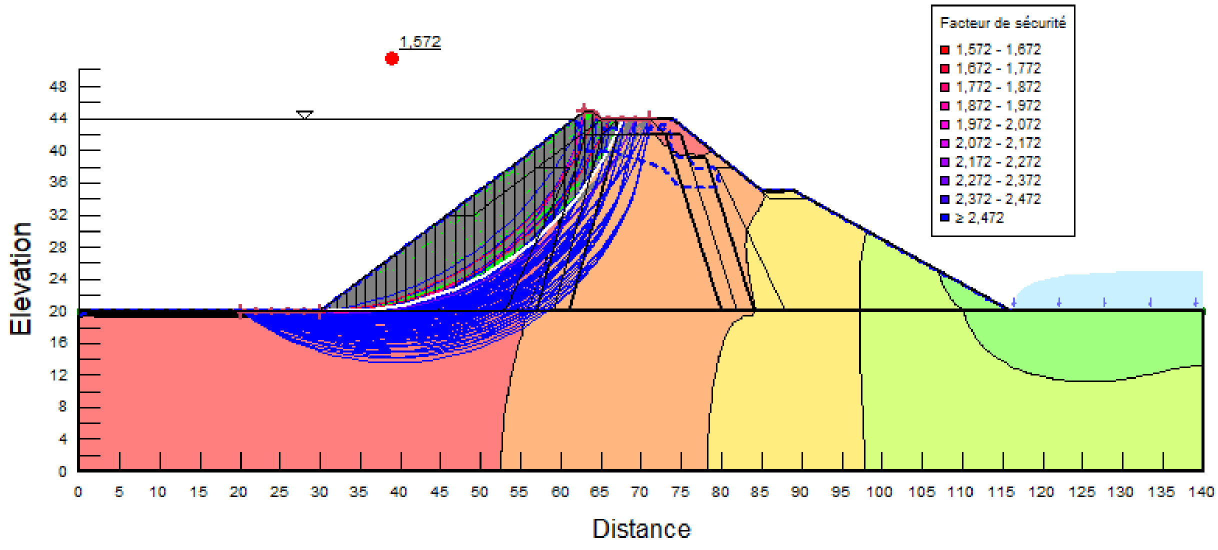

Fig 36. Critical slope stability of earthen dam of Songloulou at the water level 530 m

For the study of cases of earth dams in operation the method exposed in this paper is commonly used [50-51]. These principles were recently published in American Journal of Construction and Building material [52]. The horizontal displacement results are consistent with those recorded in the direct and indirect pendulums in the middle of the, i.e. 6 to 8 cm. In the growing desire to improve their safety, the integration of numerical investigations in the overall assessment of earthen dam is becoming essential. From the modeling, we have obtained safety factors in slope shear failure greater 1.5 for water levels in the reservoir of 528 and 530 m, reflecting the overall stability of the structure under hydraulic load.

6. Conclusion

In this paper, the prediction qualities of each of the models unequivocally indicate a preference for the ANFIS model. However, when drawing conclusions from such a study, some caution should be exercised. Indeed, all comparisons between the HST and ANFIS model are based on the inseparable data sets from the Songloulou large earthen dam. To validate our model, i.e. to know if it is capable of detecting and anomaly on the dam, it would be necessary to calibrate it using a set of data from a dam shows that the sealing layer separating the upstream part and the downstream part works and protects the downstream from the upstream well, however at the level of the spillway dam there is a significant increase from the piezometric level over time, which shows an increase in the amount of water infiltrated over the years. The safety, security and reliability of dams depend on the lives of thousands of people, so we must be careful not to act hastily in this area. Consequently, it seems to us preferable to model each dam individually and to find in each case an adequate model, for certain structures, the classis model will certainly be more adequate, while for others, the ANFIS model will lead to better results both for the treatment of pressures and displacements. The introduction of the ANFIS model expands the range of models available to perform the necessary control and prediction work on dams. This model also offers an alternative to the classis model when the latter reveals some weaknesses and allows two different models to be used in complementary way. To predict the overall behaviour of the structure, it necessary to use numerical modeling in order to assess all the inseparable parameters (displacements, pressures, seepages, leak rates). On the GEOSTUDIO modeling, the results of global displacement according to the level of the reservoir of the dam show that it remains stable as a whole. All horizontal displacements are less than 20 cm. In an advanced monitoring process, we used the auscultation data to have a realistic modeling. The horizontal displacement results are consistent with those recorded in the direct and indirect pendulums in the middle of the, i.e. 6 to 8 cm. In the growing desire to improve their safety, the integration of numerical investigations in the overall assessment of earthen dam is becoming essential. These investigations make it possible to have the overall behavior of the structure according various design analysis criteria. From the modeling, we have obtained safety factors in slope shear failure greater 1.5 for water levels in the reservoir of 528 and 530 m, reflecting the overall stability of the structure under hydraulic load.

In this paper, an analysis of auscultation devices based on artificial neural networks is implemented. The method adapts well to variations in the parameters influencing the

measurement tools and does not require ongoing recalibration during the lifetime of the structure. Unlike the HST method based on data recorded during a period (analysis period), neural networks use the entire data recorded during operation and improve their efficiency over time. The auscultation results and their analyses were backed up by numerical modeling of the earth dam making it possible to have its behavior according to the mechanical parameters of the materials taken from the geotechnical structure and the level of the hydraulic head. These results are in agreement with observations on the site. The results of the statistical study and Finite Element Modeling are in agreement with those of recent research throughout the world [10-26 ; 36-46 ; 50-52] for the analysis of the same phenomena.

Data Availability

The data used to support the findings of this study are available from the corresponding author upon request.

Conflicts of interest

The authors confirm that there are no conflicts of interest associated with this publication and there has been no significant financial support for this work that could have influenced its outcome.

Acknowledgements

Several people were involved in obtaining the results from this paper; there are very many of them and we cannot name them. Find our warm thanks. The authors acknowledge particularly the ENEO Cameroon, and Geotechnical's Laboratories and Engineering's Offices of Cameroon for the facilities and support to perform the experiments.

Author Contributions

Conceptualization: Zoa Ambassa, Jean Chills Amba.

Data curation: Zoa Ambassa, Bodol Momha Merlin, Djopkop Kouanang Landry.

Methodology: Zoa Ambassa, Bodol Momha Merlin, Jean Chills Amba.

Project administration: Jean Chills Amba, Nzengwa Robert

Resources: Mbongo Pascal Adrien.

Software: Zoa Ambassa, Bodol Momha Merlin, Djopkop Kouanang Landry.

Supervision: Nzengwa Robert.

Validation: Zoa Ambassa, Jean Chills Amba, Bodol Momha Merlin.

Writing – original draft: Zoa Ambassa, Jean Chills Amba.

Writing – review & editing: Zoa Ambassa, Jean Chills Amba.

References

1. Bonelli S, Felix F, Journeni R, Carlier D. Auscultation des barrages en terre-Modélisation de l'effet pluie, colloque scientifique EC'97, Strasbourg, 22-23 mai, 1997.

2. Royet P. La surveillance et l'entretien des petits barrages. Éditions du Cemagref. 2006.

3. Lautrin D. Vieillissement et réhabilitation des petits barrages en terre. Éditions du Cemagref. 2003, 240 p.

4. Anton S, Pougatsch H. Les barrages-du projet à la mise en service. *Presses Polytechniques et Universitaires Romandes (PPUR),* Ecole Polytechnique Fédérale de Lausanne. 2011, 714p.

5. Bourdarot E, Carrère A, J, Hoonakker M. Apports combinés de l'auscultation et de la modélisation pour l'analyse et la compréhension des barrages, XXe Congrès CIGB. - Beijing, 2000.

6. Bonelli S, Royet P. Delayed response analysis of dam monitoring data, ICOLD European Symposium, Geiranger, Norway, 25-27 June 2001.

7. Bonelli S. Delayed response analysis of dam monitoring data. Edité par le Cemagref, juillet 2007.

8. Bonelli S, Félix H, Tourment R. Fiabilité des matériaux et des structures, Interprétation des mesures d'auscultation des barrages par régression linéaire multiple « HST », 2e conférence nationale JN-FIAB'98. 1998.

9. Plancke V. Auscultation des barrages, logiciel de traitement statistique des mesures, mémoire ENIIRS, Cemagref. (1997).

10. Zoa A, Amba J, C, Mbelen F, X. Monitoring and numerical modeling of the full scale experimental embankment on soft Douala clays of Cameroon. *International Journal of Civil Engineering and Technology,* volume 11, no. 7. 2020.

11. Dardanelli G, La Loggia G, Perfeti N, Fulvio C, Luigi P, A. Monitoring displacements of an earthen dam using GNSS and remote sensing. *Remote sensing for Agriculture, Ecosystems, and Hydrology XVI 9239*, pp.574-589. 2014.

12. Gikas V, Sakellariou M. Settlement analysis of the Mornos earth dam (Greece): Evidence from numerical modeling and geodetic monitoring. *Engineering Structures 30 (11),* pp. 3074-3081. 2008.

13. Gikas V, Sakellariou M. Horizontal deflection analysis of a large earthen dam by means of geodetic and geotechnical methods. *13th FIG International symposium on deformation measurements and analysis and 4th IAG symposium on geodesy for geotechnical and structural engineering*. 2008.

14. Szostack-Chrzanowski A, Massiéra M. Relation between monitoring and design aspect of large earth dams; *Proceedings, 3rd IAG Symposium of Geodesy for Geotechnical and Structural Engineering and 12th FIG symposium on Deformation measurements*. Baden Australia. 2006.

15. Mouyeaux A, Carvajal C, Bressolette P, Peyras L, Breul P, Bacconnet C. Probabilistic stability analysis of an earth dam by Stochastic Finite Element Method based on field data. *Computers and Geotechnics*, *101*, 34-47. 2018.

16. Guo X, Dias D, Carvajal C, Peyras L, Breul P. Reliability analysis of embankment dam sliding stability using the sparse polynomial chaos expansion. *Engineering Structures*, *174*, 295-307. 2018.

17. Bal L, Buyle-Bodin F. Artificial neural network for predicting drying shrinkage of concrete. *Construction and Building Materials*, *38*, 248-254. 2013.

18. Pang R, Xu B, Kong X, Zou D, Zhou Y. Seismic reliability assessment of earth-rockfill dam slopes considering strain-softening of rockfill based on generalized probability density evolution method. *Soil Dynamics and Earthquake Engineering*, *107*, 96-107. 2018.

19. Campos A, Lopez C, M, Blanco A, Aguado A. Effects of an internal sulfate attack and an alkali-aggregate reaction in a concrete dam. *Construction and Building Materials*, *166*, 668-683. 2018.

20. Doghai L, Bo C, Yugang L, Denghua z. Automatic control and real-time monitoring system for earth-rock dam material truck watering. *Automation in Construction 30*, pp.70-80. 2013.

21. Cejka F, Benes V, Glac F, Boukalova Z. Monitoring of seepages in earthen dams and levees. *International Journal of Environmental Impacts* 1 (3), pp. 267-278. 2018.

22. Krzysztof R, Bonelli S. Thermal seepage monitoring in the earth dams with impulse response function analysis model. *Na*. 2010.

23. Inaudi D, Church J. Paradigm shifts in monitoring levees and earth dams distributed fiber optic monitoring systems. *31st USSD Annual Meeting & Conference*, San Diego, California, USA. 2011.

24. Pagano L, Sica S. Earthquake early warning for earth dams: Concepts and objectives. *Natural hazards*, 66 (2), 303-318. 2013.

25. Di Martire D, Iglesias R, Monells D, Centolanza G, Sica S. Comparison between differential SAR interferometry and ground measurements data in the

displacement monitoring of the earth-dam of Conza della Campania (Italy). *Remote sensing of environment, 148*, pp. 58-69. 2014.

26. Andreini M, Gardoni P, Pagliara S, Sassu M. Probabilistic models for the erosion rate in embankments and reliability analysis of earth dams. *Reliability Engineering & System Safety*, *181*, 142-155. 2019.

27. Guillemot T, Lino M, Nzali E. Diagnostic et mise en sécurité du barrage de Songlou au Cameroun vis-à-vis des désordres liés à l'alcali-réaction. Colloque technique, Modernisation des barrages, CFBR. 2013. 10p.

28. Ferry S, William G. Méthodes d'analyse et de surveillance des déplacements observés par le moyen de pendules dans les barrages. VIth International Congress on Large Dams, New-York, vol. II, ICOLD. 1958, pp.1179-1201.

29. Touzet C. Les réseaux de neurones artificiels, introduction au connexionnisme: cours et exercices et travaux pratiques: Collection de l'EERIE. 1992.

30. Abrahart R, J, See L, M, Kneale P, E. Neural network for hydrological modelling: Taylor & Francis e-Library. 2005.

31. Godjevac J. Idées nettes sur la logique floue. *Presses Polytechniques et Universitaires Romandes*. 1999.

32. Chuen C. Fuzzy Logic in Control Systems: Fuzzy Logic Controller, part I. IEEE Transactions on System Mann. And cyri Rsetrs, Vol. 20, No. 2, March/April 1990, pp: 405-417.

33. Chuen C. Fuzzy Logic in Control Systems: Fuzzy Logic Controller, part II" IEEE Transactions on System Mann. And cyri Rsetr, Vol. 20 No. 2, March/April 1990 pp: 419-435.

34. Rafael A, Gouriveau M, Zerhouni N. Pronostic de défaillances: maitrise de l'erreur de prédiction, Manuscrit auteur, publié dans 7ème Conférence Internationale de Mobilisation et Simulation, MOSIM'08, Paris, France. 2008.

35. MATLAB Software Development Tools. 2019

36. Najafzadeh M, Tafarojnoruz A. Evaluation of neuro-fuzzy GMDH-based particle swarm optimization to predict longitudinal dispersion coefficient in rivers, *Environmental Earth sciences*, vol.75. 2016.

37. Najafzadeh M, Saberi-Movahed F, Sarkamaryan S. NF-GMDH Based Self-Organized Systems to Predict Bridge Pier Scour Depth Under Debris Flow Effects, *Marine Georesources and Geotechnology*, vol.36 (5). 2017.

38. Najafzadeh M, Etemad-Shahidi A, Lim S. Y. Scour prediction in long contractions using ANFIS and SVM, *Ocean Engineering*, 111. 2015, pp. 128-135.

39. Najafzadeh M, Azamathulla H. Neuro-Fuzzy GMDH to predict the Scour Pile Groups due to Waves, *Journal of Computing in Civil Engineering*, ASCE. 2014, pp. 1-9.

40. Najafzadeh M, Sattar A. M. A. Neuro-Fuzzy GMDH Approach to Predict Longitudinal Dispersion in Water Networks, *Water Resource Management*. 2015, 16 p.

41. Samarasinghe J. T, Basnayaka V, Gunathilake M. B, Azamathulla H. Rathnayake M, U. Comparing Combined 1D/2D and 2D Hydraulic Simulations

Using High-Resolution Topographic Data: Examples from Sri Lanka – Lower Kelani River Basin, *Marine Hydrology*, MPDI, vol.9 (39). 2022.

42. Ahmadpour R, Sarkadeh H, Azamathulla H. Mathematical simulation of air-water flow along Ski Jump Jet, *Water Supply*, vol.0 (0). 2021.

43. Najafzadeh M. Neurofuzzy-Based GMDH-PSO to Predict Maximum Scour Depth at Equilibrium at Culvert Outlets, *Journal of Pipeline System Engineering Practice*, ASCE. 2015, pp. 1-6.

44. Amininia K, Saghebian S.M. Uncertainty analysis of monthly river flow modeling in consecutive hydrometric stations using integrated data-driven models, *Journal of Hydroinformatics*. 2021, 17 p.

45. Ghasempour R, Roushangar K, Azamathulla H. M, Alizadeh F. Uncertainty Analysis of Flow Resistance Modeling In Sewer Pipe With Different Bed Conditions Using KELM Method, *Research Square*. 2021, 14 p.

46. Zoa A, Amba J.C. Assessment of stiffness and strength parameters for the soft soil Model of clays of Cameroon, *Hindawi, International Journal Advances in Civil Engineering,* volume 2020.

47. GEOSTUDIO©. The modeling software for geo-engineers and earth scientists. 2018.

48. Davis R. O, Selvadurai A.P.S. *Plasticity and Geomechanics*, Cambridge University Press. 2002.

49. Hill R. *The Mathematical Theory of Plasticity*, Oxford University Press, U.S.A. 1998.

50. Mao W-L, Shu Gu Y-M, Zhang X-L, Zhang Z. Horizontal seepage failure model and experimental study of damaged sidewalls of seams between geotube dam tubes, *PLoS ONE (4): e0231624.https:/doi.org/10.1371/journal.pone.0231624*, 2020.

51. Mirostaw-Swiatek D, Popielski P, Sliwinski P, Cwalina T. S. Analysis of factors influencing levee safety using the DEMATEL method, *PLoS ONE 16(9): e0255755. https://doi.org/10.1371/journal.pone.0255755*, 2021.

52. Amba J. C, A. Zoa A, Offolé F, Essola D, Bodol Momha M, Nzengwa R,. Mbongo P. A. Determination of Water Level on the Reservoir of the Earthen Dam of Songloulou Based on the Artificial Neural Network Method and Assessment of the Stress and Displacements, *American Journal of Construction and Building Material*, 2022.
