# Supplementary material for: Advanced monitoring and numerical modelling of the stability, safety and reliability indicators of the earthen dam of Songloulou (Cameroon)
Source: PLoS One. 2023 Oct 11;18(10):e0292804. doi: 10.1371/journal.pone.0292804 (PMC10566693; doi:10.1371/journal.pone.0292804)
Supplement: Attachment — Submitted filename: Leter for Academic Editor_Response to Reviewers.pdf [file pone.0292804.s004.pdf]

September, 19, 2023

To: Professor Ahad Javanmardi

Academic Editor of PLOS ONE

Subject: Submission of manuscript revised 2

Reference: PONE-D-23-03305

Dear Professor

Please find enclosed a revised version of our paper entitled: “**Advanced Monitoring and Numerical Modelling of the Stability, Safety and Reliability Indicators of the Earthen Dam of Songloulou (Cameroon)**”.

General comments

Academic Editor:

We appreciate the Academic Editor and reviewer’s comments and have proceeded with all relevant changes. We have replied point-by-point to all comments submitted by the referee. New comments are highlighted in red color in the file labeled ‘Revised Manuscript with Track Changes’.

Specific comments

All modification has been inserted in the paper in red color in the file labeled ‘Revised Manuscript with Track Changes’.

Figure 1 (original manuscript, p. 5 ; line 130 and 131) has been deleted in the revised version. The numbers of other figures have been replaced in red color in the file labeled ‘Revised Manuscript with Track Changes’.

The main advantages of the ANFIS model in practical engineering applications are the prediction the evolution of the critical phenomena as a function of time and environmental conditions and the failure mechanism of structures. (see, p.4 ; line 119 to 122 of the file labeled ‘Revised Manuscript with Track Changes’). This comment is not mentioned in the original manuscript, p. 5 ; line 145.

The recent references have been added in the introduction part. (see references 27 to 29, p. 3, line 85 to 86 of the file labeled ‘Revised Manuscript with Track Changes’).

The lack data from altimetric measurements does not make it possible to have settlments or swelling on the top of the dam according to the seasons. This will be the subject of future

investigations. (see p. 33, lines 631 to 633 of the file labeled ‘Revised Manuscript with Track Changes’). These comments are not mentioned in the original manuscript, p. 33, line 604.

- *There are some grammatical mistakes in the manuscript. Please check the English of the manuscript, thoroughly.*

Dear Academic Editor, many sentences in this paper have been modified and rewritten in the text in red color (see the file labeled ‘Revised Manuscript with Track Changes’).

- *Figures should come after being cross-referred to in the text. All the figures and tables should be cited in the text.*

All figures and tables of this paper have been cited in the text in this revised version (see the file labeled ‘Revised Manuscript with Track Changes’).

- *Please provide a main caption for Figure 6.*

The main caption for figure 5 (figure 6 for the original manuscript, p. 7 ; line 169 to 179) has been modified and replaced in the text (see p. 6, lines 145 and 146 of the file labeled ‘Revised Manuscript with Track Changes’).

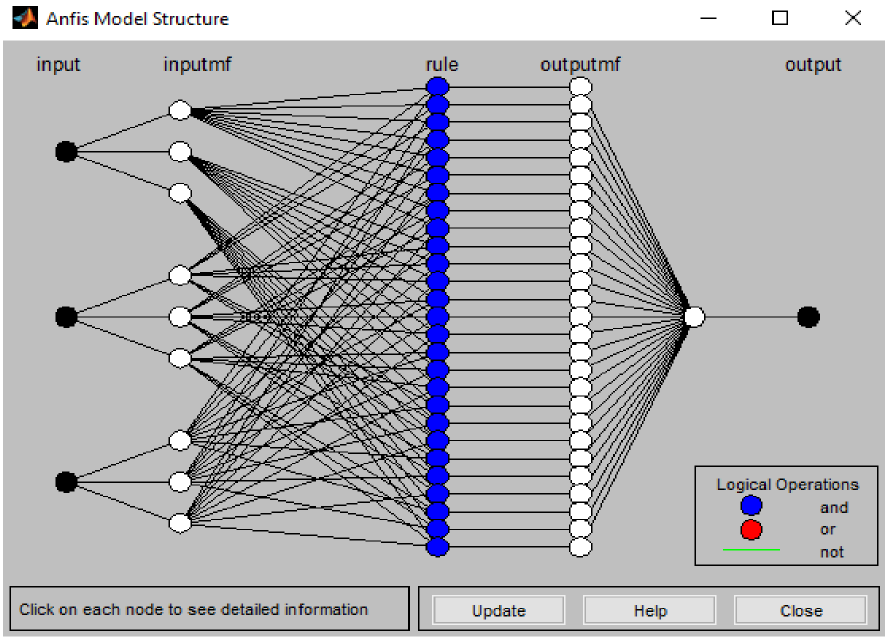

Fig 5. Architecture of the ANFIS network in MATLAB software

- *The quality of the figures is poor. The legends are not clear and in some of the figures, the title axis is in French or other languages.*

The Clarity of the figures has been treated. The legend and the titles of the axes have been rewritten in english (see the file labeled ‘Revised Manuscript with Track Changes’).

- *In Table 5, please add a title for the D and Da columns that clear the type of these parameters.*

The definition for D and Dα have been inserted in the title of the table 5.

D is the maximum difference in absolute value between the observed cumulative proportions and the simulated cumulative proportions; Dα: the critical value. (see p. 11, lines 230 to 232 of the file labeled 'Revised Manuscript with Track Changes'). This comment is not mentioned in the original manuscript, p. 12 ; line 272.

- *The detail of the finite element model (section 5) is insufficient. Please provide more details of the FEM.*

The detail of finite element model in section 5 have been inserted. (see p. 24, lines 455 to 468 of the file labeled 'Revised Manuscript with Track Changes'). These old comments are not mentioned in the original manuscript, p. 25 ; line 493.

"The SIGMA/W module of GEOSTUDIO was used to obtain the stresses, strains and displacements of the Finite element Model. The mesh of the model has 6210 triangular elements with 3 nodes. The SLOPE module of GEOSTUDIO was used to assess the slope stability of the earth dam. This module uses the method of resolution by limit equilibrium. Bishop's method was used. The model is subject to loading relative to the weight of the materials and the water pressure from the front side".

- *Please provide the definition for the notations used in Table 7.*

The definition of the notation in table 7 have been inserted. (see p. 25, line 471 of the file labeled 'Revised Manuscript with Track Changes'). These definitions are not mentioned in the original manuscript, p. 26 ; line 502.

Table 7. Parameters of subsoil and earth materials

Material	Unit weight of soil: γ_{unsat} (kN/m^3)	Saturated unit weight of soil: γ_{sat} (kN/m^3)	Cohesion in terms of effective stress: c' (kPa)	Effective angle of shearing resistance: Φ' (°)	Young's modulus (MPa)	Poisson's ratio (-)	Coefficient of permeability (m/s)
Core	18	19	15	32	74	0.3	1.15741E-14
Rip-Rap	22	24	0	40	80	0.3	1.15741E-12
Filtered	19	22	0	40	400	0.3	2.3148E-12
Rockfill	22	24	0	45	800	0.3	1.15741E-12
Rock foundation	26	26	1000	45	5000	0.3	5.78704E-14

- *There is no validation or calibration of the FE model. Please elaborate on the accuracy of the model.*

To ensure the reliability of the results, the meshing of the model was carried out according to the Mestat recommendations for earth dams [53-54]. In order to validate our GEOSTUDIO calculation program, the results of this part were compared with those of other software commonly used in research and industrial environments. Two other

softwares were used. These results concern horizontal displacements with the Finite element code Cast3M [55] and Finite element code Plaxis [56] at retention level 528 and 530 m of the dam. The horizontal displacements calculated are: 11.68 cm; 12 cm; 11.90 cm (at retention level 528 m) and 20 cm; 19 cm and 19.47 cm (at retention level 530 m) respectively for GEOSTUDIO, Cast3M and Plaxis softwares. (see p. 24, lines 461to 464 ; p. 25, lines 473 to 479 and p. 28, line 510 to p. 30, line 540 of the file labeled 'Revised Manuscript with Track Changes'). These comments and figures of Cast3M and Plaxis modelling are not mentioned in the original manuscript, p. 28 ; line 529.

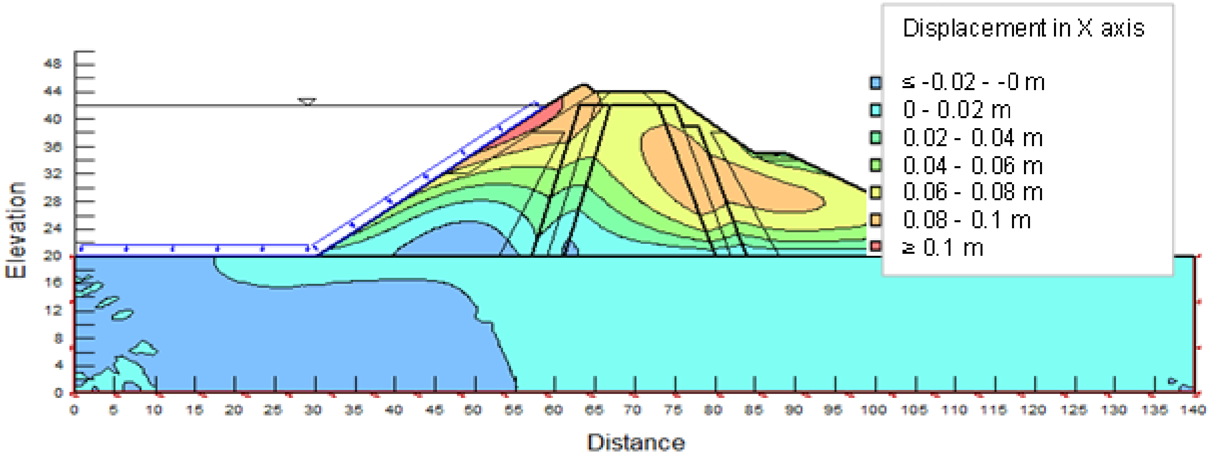

Fig 28. Horizontal displacements at the water level 528 m obtained by GEOSTUDIO software

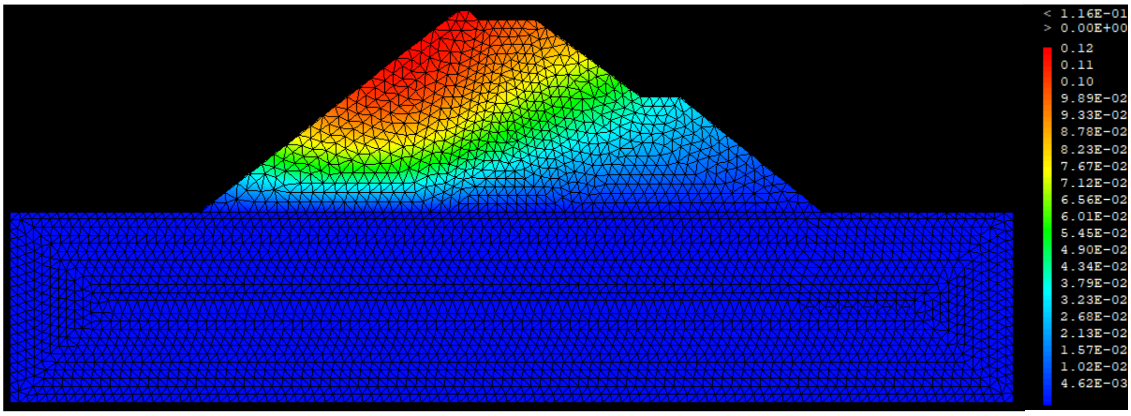

Fig 29. Horizontal displacements at the water level 528 m obtained by Cast3M software (this figure of Cast3M modelling is not mentioned in the original manuscript, p. 28; line 511).

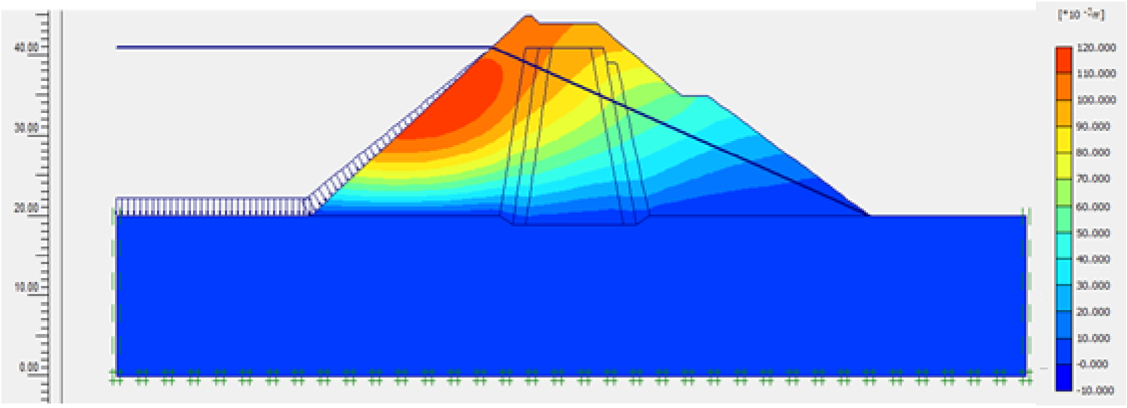

Fig 30. Horizontal displacements at the water level 528 m obtained by Plaxis software (this figure of Plaxis modelling is not mentioned in the original manuscript, p. 28; line 514).

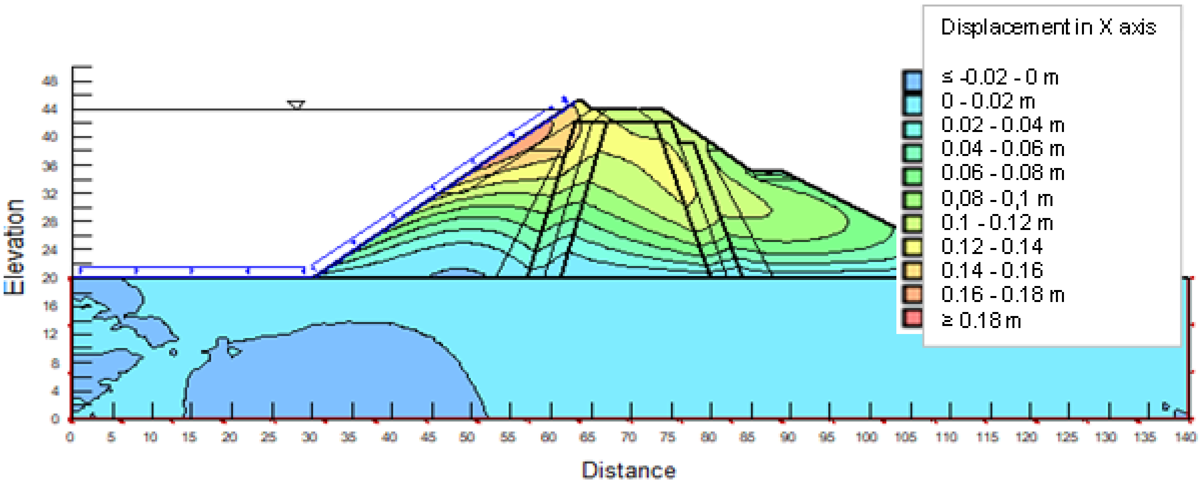

Fig 34. Horizontal displacements at the water level 530 m obtained by GEOSTUDIO software

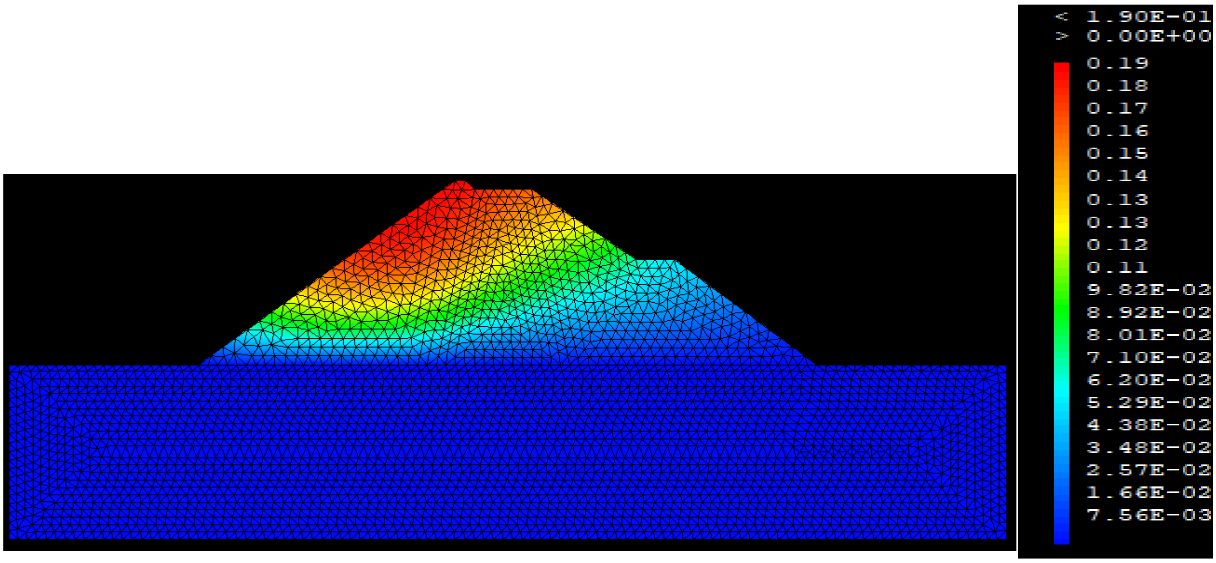

Fig 35. Horizontal displacements at the water level 530 m obtained by Cast3M software (this figure of Cast3M modelling is not mentioned in the original manuscript, p. 30; line 536).

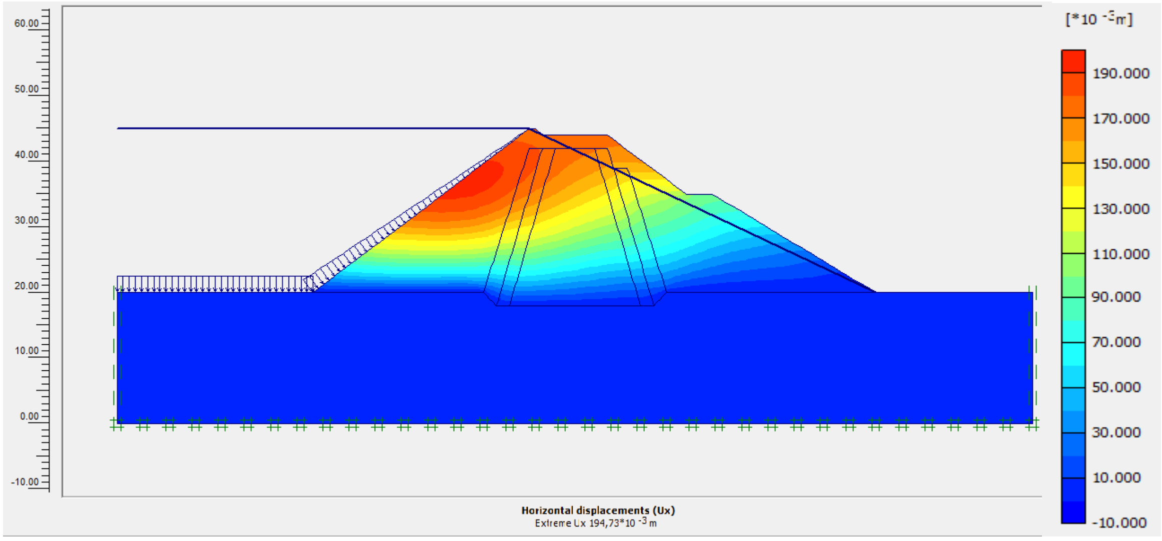

Fig 36. Horizontal displacements at the water level 530 m obtained by Plaxis software (this figure of Plaxis modelling is not mentioned in the original manuscript, p. 30; line 539).

- *Please quantify results in the conclusion section and write them point-wise.*

Monitoring data did not concern the measurements of vertical displacements in the dam. These vertical displacements in the model are given as a complement to know the global behaviour of the dam. Only the calculated horizontal displacements are compared to the results of the pendulums.

The water level of the dam fluctuates between the retention level 522 m and 528 m. The monitoring results and their analyses were backed up by numerical modelling of the earth dam making it possible to have its behaviour according to the mechanical parameters of the materials taken from the geotechnical structure and the level of the hydraulic head.

The horizontal displacements measured on the dam are around 7.4 cm (see table 8). The average horizontal displacements obtained by numerical modelling at retention level 522 and 528 m is 8.84 cm. These results are consistent with the behaviour of the structure. These results are in agreement with observations on the site. The modelling provides other useful information such as the vertical stresses prevailing at any point in the model.

Table 8. Summary of modelling results and pendulum measurements

**Retention level (m)**	**Ux : Horizontal displacement (cm)**	**Uy : Vertical displacement (cm)**	**σyy: Vertical total stress (kPa)**	**Facteur of safety of slope**	**Pendulum P1D measurements: Ux (cm)**	
					**Upstream - Downstream**	**left-right**
522	6	74	900	0.219		
528	11.68	80	912	1.772	7.44	7.11
530	20	80	920	1.572		

(see p. 26, lines 495 to 498 ; p.32, line 547 and p. 34, lines 621 to 629 of the file labeled 'Revised Manuscript with Track Changes'). These comments and table 8, are not mentioned in the original manuscript, pp. 31-32.

The references have been modified and replaced in new version of the paper. (see p. 39, lines 805 to 815 for four new references 53 to 56 of the file labeled 'Revised Manuscript with Track Changes'). These references are not mentioned in the original manuscript, p. 37.

References

53. Mestat P. Maillage d'éléments finis pour les ouvrages géotechniques. Conseils et recommandations, *Bulletin des laboratoires des ponts et chaussées*, no. 212, pp. 39-64, 1997.

54. Mestat P, Humbert P, Dubouchet A. Recommandations pour la vérification des modèles d'éléments finis en géotechnique, *Bulletin des laboratoires des ponts et chaussées*, no. 229, pp. 33-51. 2000.

55. Cast3M©, Cast3M is a research FEM environment; its development is sponsored by the French Atomic Energy Commission, see web site: http://www/cast3m.cea.fr/, 2021.

56. PLAXIS©, "Finite Element Code for Soil and Rock Analysis", *Material Models Manual*, v.8, 2018, 256 p.

Reviewer #1: September, 14, 2023 :

The author partially solved the problems raised by the reviewers, which is worthy of recognition. However, there are still some important issues in the manuscript that have not been fully explained. The current manuscript form is suggested to be slightly revised before it can be published by PLOS ONE. The following important problems should be fully solved.

- *1. The basic model (HST) tends to generalize the system, and approximates the behavior of the piezometer more or less well until 2015 when the peaks become larger and larger, from then on the HST model does not can't keep up anymore. There are then notable differences between the two models. The reason for this remarkable difference should be explained.*

An assumption of the HST method is that «errors are distributed according to the normal distribution $\varepsilon\_i \equiv N(0, \sigma\_\varepsilon)$ » but in the case of the piezometer M1-1, from 2015, the empirical distribution of residues no longer fits with the Normal distribution. (see page 13, line 238 to line 241 of the file labeled 'Revised Manuscript with Track Changes'). These comments are not mentioned in the original manuscript.

- *2. In the numerical simulation of the earth dam water level, we should give a comparative analysis with similar research. At the same time, a philosophical explanation of the distribution of stress and displacement should be given.*

Numerical modelling was used to determine the stress and displacement in the structure. These stresses and displacements make it possible to prevent the failure mechanisms and collapse according to the functional requirements relating to the structure. The iso-values of the vertical stress (Fig 26, 32 and 38) show a concentration of the maximum values at the bottom of the model to the right of the core size. This concentration is consistent with the theoretical distribution mechanism of vertical stresses, $\sigma_{yy}=\Sigma\gamma_i \times h_i$ (γ_i: unit weight of soil for layer i and h_i, thickness of layer i). With parameters from Table 7 and taking into account the heights of the different layers, the theoretical vertical stress to the right of the core is $\sigma_{yy}= (26 \times 20 + 18 \times 23.5) = 943$ kPa. This value is close to those obtained in Fig 26, 32 and 38.

The iso-values of the vertical displacements along the y-axis are consistent with the vertical stresses: maximum displacement at the top of the model and no displacement at the bottom of the model (Fig 25, 31 and 37) to respect the boundaries conditions of the displacements at the bottom of the model.

The iso-values and shape of the horizontal displacements (Fig 24, 28, 29, 30, 34, 35 and 36) are consistent with the sliding failure mechanism of such a structure (circular sliding to the left of the model) (Fig 27, 33 and 39). This sliding mechanism and associated values depend on the level of water retention in the earthen dam. The slip safety factors are greater than 1.5 guarantee the stability of the structure. Nevertheless, precautions should be taken by the managers of this dam when the water level is less than or equal to the 522m rating to ensure its stability. The numerical modelling results found in this paper are consistent with those found by Mouyeaux et al. [15] who did stochastic numerical modelling on an earthen dam in France using the Cast3M calculation code. In his model the iso-values of maximum stresses were at the bottom of the model. The sliding collapse mechanism is circular. In their study, several slip safety factors were evaluated based on the variation in water retention levels in the dam and the mechanical parameters of the materials. (see page 32, line 556 to page 33, line 580 of the file labeled 'Revised Manuscript with Track Changes'). These comments are not mentioned in the original manuscript.

- *3. The clarity of the pictures in Figures 25-36 should be improved, which is more convenient for readers to read. For example: increase the font size in all the figures to enhance the readability.*

The Clarity of the pictures in Figures 25-36 (for first version) and Figures 24-39 for revised version has been treated. The legends have been rewritten. (see the file labeled 'Revised Manuscript with Track Changes' page 25, line 484 to page 32, line 546).

- *4. What are the lessons learnt from the conclusions other than the observation? Is this finding original to this paper or is it a validation of a previously established notion/fact?*

The Anfis method is a learning process that improves over time, which minimizes its error. The HST method is based on an analysis of data in a given time interval; on the other hand, even if its estimator is BLUE (Best Linear Unbiased Estimator), the predictions may turn out to be false as soon as the flow conditions change. It is therefore important, before using this method, to carry out a type 1 analysis (Check of data conformity). (see the file labeled 'Revised Manuscript with Track Changes' page 33, line 598 to line 603).

Final comments

We appreciate all suggestions and comments provided by the referee. If some of our responses are still unclear, please let us know at your earliest convenience.

The funders had no role in study design, data collection and analysis, decision to publish, or preparation of the manuscript.

The authors received no specific funding for this work.

The authors have declared that no competing interests exist.

Yours sincerely,

Dr ZOA AMBASSA
