# Supplementary material for: Advanced monitoring and numerical modelling of the stability, safety and reliability indicators of the earthen dam of Songloulou (Cameroon)
Source: PLoS One. 2023 Oct 11;18(10):e0292804. doi: 10.1371/journal.pone.0292804 (PMC10566693; doi:10.1371/journal.pone.0292804)
Supplement: Attachment — Submitted filename: Leter for Academic Editor_Response to Reviewers.docx [file pone.0292804.s002.docx]

May, 17, 2023

**To**: Professor Ahad Javanmardi

Academic Editor of PLOS ONE

**Subject:** Submission of manuscript revised

**Reference:** PONE-D-23-03305

Dear Professor

Please find enclosed a revised version of our paper entitled: “**Advanced Monitoring and Numerical Modelling of the Stability, Safety and Reliability Indicators of the Earthen Dam of Songloulou (Cameroon)**”.

**General comments**

Academic Editor:

We appreciate the Academic Editor and reviewer’s comments and have proceeded with all relevant changes. We have replied point-by-point to all comments submitted by the referee. New comments are highlighted in red in the revised manuscript.

**Specific comments**

All modification has been inserted in the paper in red color.

Figure 1 has been deleted in the revised version. The numbers of other figures have been replaced.

The main advantages of the ANFIS model in practical engineering applications are the prediction the evolution of the critical phenomena as a function of time and environmental conditions and the failure mechanism of structures. (see line 199 to 122).

The recent references have been added in the introduction part. (see references 27 to 29).

The lack data from altimetric measurements does not make it possible to have settlments or swelling on the top of the dam according to the seasons. This will be the subject of future investigations.

- *There are some grammatical mistakes in the manuscript. Please check the English of the manuscript, thoroughly.*

Dear Academic Editor, many sentences in this paper have been modified and rewritten in the text in red color.

- *Figures should come after being cross-referred to in the text. All the figures and tables should be cited in the text.*

All figures and tables of this paper have been cited in the text in this revised version.

- *Please provide a main caption for Figure 6.*

The main caption for figure 5 (figure 6 for first version) has been modified and replaced in the text.

**Fig 5.** Architecture of the ANFIS network in MATLAB software

- *The quality of the figures is poor. The legends are not clear and in some of the figures, the title axis is in French or other languages.*

The Clarity of the figures has been treated. The legend and the titles of the axes have been rewritten in english.

- *In Table 5, please add a title for the D and Da columns that clear the type of these parameters.*

The definition for D and Dα have been inserted in the title of the table 5.

D is the maximum difference in absolute value between the observed cumulative proportions and the simulated cumulative proportions; Dα: the critical value)

- *The detail of the finite element model (section 5) is insufficient. Please provide more details of the FEM.*

The detail of finite element model in section 5 have been inserted. See line 454 to 468.

The SIGMA/W module of GEOSTUDIO was used to obtain the stresses, strains and displacements of the Finite element Model. The mesh of the model has 6210 triangular elements with 3 nodes.

The SLOPE module of GEOSTUDIO was used to assess the slope stability of the earth dam. This module uses the method of resolution by limit equilibrium. Bishop’s method was used. The model is subject to loading relative to the weight of the materials and the water pressure from the front side.

- *Please provide the definition for the notations used in Table 7.*

The definition of the notation in table 7 have been inserted.

**Table 7.** Parameters of subsoil and earth materials

| Material | Unit weight of soil: γ_unsat_ (kN/m^3^) | Saturated unit weight of soil: γ_sat_ (kN/m^3^) | Cohesion in terms of effective stress: c’ (kPa) | Effective angle of shearing resistance: Φ’ (°) | Young’s modulus (MPa) | Poisson’s ratio (-) | Coefficient of permeability (m/s) |
| --- | --- | --- | --- | --- | --- | --- | --- |
| Core | 18 | 19 | 15 | 32 | 74 | 0.3 | 1.15741E-14 |
| Rip-Rap | 22 | 24 | 0 | 40 | 80 | 0.3 | 1.15741E-12 |
| Filtered | 19 | 22 | 0 | 40 | 400 | 0.3 | 2.3148E-12 |
| Rockfill | 22 | 24 | 0 | 45 | 800 | 0.3 | 1.15741E-12 |
| Rock foundation | 26 | 26 | 1000 | 45 | 5000 | 0.3 | 5.78704E-14 |

- *There is no validation or calibration of the FE model. Please elaborate on the accuracy of the model.*

To ensure the reliability of the results, the meshing of the model was carried out according to the Mestat recommendations for earth dams ­[50-51]. In order to validate our GEOSTUDIO calculation program, the results of this part were compared with those of other software commonly used in research and industrial environments. Two other softwares were used. These results concern horizontal displacements with the Finite element code Cast3M [52] and Finite element code Plaxis [53] at retention level 528 and 530 m of the dam. The horizontal displacements calculated are: 11.68 cm; 12 cm; 11.90 cm (at retention level 528 m) and 20 cm; 19 cm and 19.47 cm (at retention level 530 m) respectively for GEOSTUDIO, Cast3M and Plaxis softwares (see Fig 28 to 30 and Fig 34 to 36).

**Fig 28.** Horizontal displacements at the water level 528 m obtained by GEOSTUDIO software

**Fig 29.** Horizontal displacements at the water level 528 m obtained by Cast3M software

**Fig 30.** Horizontal displacements at the water level 528 m obtained by Plaxis software

**Fig 34.** Horizontal displacements at the water level 530 m obtained by GEOSTUDIO software

**Fig 35.** Horizontal displacements at the water level 530 m obtained by Cast3M software

**Fig 36.** Horizontal displacements at the water level 530 m obtained by Plaxis software

- *Please quantify results in the conclusion section and write them point-wise.*

Monitoring data did not concern the measurements of vertical displacements in the dam. These vertical displacements in the model are given as a complement to know the global behaviour of the dam. Only the calculated horizontal displacements are compared to the results of the pendulums.

The water level of the dam fluctuates between the retention level 522 m and 528 m. The monitoring results and their analyses were backed up by numerical modelling of the earth dam making it possible to have its behaviour according to the mechanical parameters of the materials taken from the geotechnical structure and the level of the hydraulic head.

The horizontal displacements measured on the dam are around 7.4 cm (see table 8). The average horizontal displacements obtained by numerical modelling at retention level 522 and 528 m is 8.84 cm. These results are consistent with the behaviour of the structure. These results are in agreement with observations on the site. The modelling provides other useful information such as the vertical stresses prevailing at any point in the model.

**Table 8.** Summary of modelling results and pendulum measurements

| Retention level (m) | Ux : Horizontal displacement (cm) | Uy : Vertical displacement (cm) | σyy: Vertical total stress (kPa) | Facteur of safety of slope | Pendulum P1D measurements: Ux (cm) | |
| --- | --- | --- | --- | --- | --- | --- |
|  |  |  |  |  | Upstream -Downstream | left-right |
| 522 | 6 | 74 | 900 | 0.219 | 7.44 | 7.11 |
| 528 | 11.68 | 80 | 912 | 1.772 |  |  |
| 530 | 20 | 80 | 920 | 1.572 |  |  |

The reference have been modified and replaced in new version of the paper.

**References**

**50.** Mestat P. Maillage d’éléments finis pour les ouvrages géotechniques. Conseils et recommandations, *Bulletin des laboratoires des ponts et chaussées*, no. 212, pp. 39-64, 1997.

**51.** Mestat P, Humbert P, Dubouchet A. Recommandations pour la vérification des modèles d’éléments finis en géotechnique, *Bulletin des laboratoires des ponts et chaussées*, no. 229, pp. 33-51. 2000.

**52.** Cast3M^©^, Cast3M is a research FEM environment; its development is sponsored by the French Atomic Energy Commission, see web site: <http://www/cast3m.cea.fr/>, 2021.

**53.** PLAXIS^©^, “Finite Element Code for Soil and Rock Analysis”, *Material Models Manual*, v.8, 2018, 256 p.

**54.** Mao W-L, Shu Gu Y-M, Zhang X-L, Zhang Z. Horizontal seepage failure model and experimental study of damaged sidewalls of seams between geotube dam tubes, *PLoS ONE (4): e0231624.https:/doi.org/10.1371/journal.pone.0231624*, 2020.

**55.** Mirostaw-Swiatek D, Popielski P, Sliwinski P, Cwalina T. S. Analysis of factors influencing levee safety using the DEMATEL method, *PLoS ONE 16(9): e0255755. https://doi.org/10.1371/journal.pone.0255755*, 2021.

**56.** Amba J. C, A. Zoa A, Offolé F, Essola D, Bodol Momha M, Nzengwa R,. Mbongo P. A. Determination of Water Level on the Reservoir of the Earthen Dam of Songloulou Based on the Artificial Neural Network Method and Assessment of the Stress and Displacements, *American Journal of Construction and Building Material*, 2022.

**Final comments**

We appreciate all suggestions and comments provided by the referee. If some of our responses are still unclear, please let us know at your earliest convenience.

The funders had no role in study design, data collection and analysis, decision to publish, or preparation of the manuscript.

The authors received no specific funding for this work.

The authors have declared that no competing interests exist.

Yours sincerely,

Dr ZOA AMBASSA
